# Effect of Hybrid mono/bimetallic Nanocomposites for an enhancement of Catalytic and Antimicrobial Activities

**DOI:** 10.1038/s41598-020-59491-5

**Published:** 2020-02-13

**Authors:** Kuppan Sivaranjan, Osaimany Padmaraj, Jayadevan Santhanalakshmi, Malairaj Sathuvan, Anbazhagan Sathiyaseelan, Suresh Sagadevan

**Affiliations:** 1 Department of Chemistry, Faculty of Science and Mathematics, Universiti Pendidikan Sultan Idris, Tanjung Malim, Perak 35900 Malaysia; 2 Department of Nuclear Physics, University of Madras, Guindy Campus, Chennai, 600 025 Tamil Nadu India; 3 Department of Physical Chemistry, University of Madras, Guindy Campus, Chennai, 600 025 Tamil Nadu India; 4 Centre for Advanced Studies in Botany, University of Madras, Guindy Campus, Chennai, 600 025 Tamil Nadu India; 5 Nanotechnology & Catalysis Research Centre, University of Malaya, Kuala Lumpur, 50603 Malaysia

**Keywords:** Biocatalysis, Synthesis of graphene

## Abstract

Exploring the new catalytic systems for the reduction of organic and inorganic pollutants from an indispensable process in chemical, petrochemical, pharmaceutical and food industries, etc. Hence, in the present work, authors motivated to synthesize bare reduced graphene oxide (rGO), polyaniline (PANI), three different ratios of rGO-PANI_(80:20,__50:50, 10:90)_ composites and rGO-PANI_(80:20,__50:50, 10:90)_ supported mono (Pd) & bimetallic [Pd: Au_(1:1,__1:2, 2:1)_] nanocomposite by a facile chemical reduction method. Also, it investigated their catalytic performances for the reduction of organic/inorganic pollutants and antimicrobial activities. All the freshly prepared bare rGO, PANI, three different ratios of rGO-PANI_(80:20, 50:50,__10:90)_ composites and rGO-PANI_(80:20, 50:50,__10:90)_/Pd & Pd: Au_(1:1, 1:2,__2:1)_ nanocomposite hybrid catalysts were characterized using UV-Vis, FT-IR, SEM, FE-SEM, EDAX, HR-TEM, XRD, XPS and Raman spectroscopy analysis. Among them, an optimized best composition of rGO-PANI_(80:20)_/Pd: Au_(1:1)_ bimetallic nanocomposite hybrid catalyst exhibits better catalytic reduction and antimicrobial activities than other composites, as a result of strong electrostatic interactions between rGO, PANI and bimetal (Pd: Au) NPs through a synergistic effect. Hence, an optimized rGO-PANI_(80:20)_/Pd:Au_(1:1)_ bimetallic nanocomposite catalyst would be considered as a suitable catalyst for the reduction of different nitroarenes, organic dyes, heavy metal ions and also significantly inhibit the growth of S. aureus, S. Typhi as well as Candida albicans and Candida kruesi in wastewater.

## Introduction

In recent years, nitroaromatics, organics dyes, and heavy metal ions possess serious environmental issues, especially by releasing toxic and carcinogenic materials in wastewater, which becomes a solemn public concern on human health and safety threats^1,2^. Therefore, research efforts have been made extensively by exploring effective catalytic systems through various techniques and processes like adsorption, ion exchange, chemical precipitation, membrane-based filtration and reductive degradation, etc., for the reduction of organic/inorganic pollutants and antimicrobial activities^3–5^. Among the aforementioned techniques, reductive degradation has been considered as an efficient conventional technique to remove the toxic and carcinogenic contaminants in waste water^6–8^. Over the past decades, carbonaceous materials such as carbon nanotubes (CNT), carbon black and graphene (rGO), etc., have been used as catalyst for different applications, because of its high surface area, high electrical conductivity as a result of an efficient collection and transfer of electrons and also facilitates the complex formation with the conductive polymer or metal nanoparticles^9–12^.

As we know that, graphene is a two-dimensional thick sheets of *sp*^2^ hybridized carbon atoms arranged in a honeycomb lattice, which exhibits high mechanical, thermal, chemical and electrical properties than that of other carbon materials^13–21^. Though graphene displays better electrical conductivity, it still lacks the active surface area owed to an inevitable aggregation of rGO nanosheets, which lead to hinder the catalytic performacnes^22–31^. Hence, researchers put tremendous effort into this present work to develop rGO composites with the conductive polymer and mono & bimetallic nanoparticles for preventing the aggregation of rGO sheets and also enhance the active surface area, which could be effectively satisfied for catalytic applications^32,33^. Among the various polymers, polyaniline (PANI) has been considered as attractive conducting polymer, because of its unique features like high conductive properties, low cost, easier synthesis processes, environmental stability and biocompatability^34^. However, it has certain drawbacks such as swelling, poor mechanical stability and electron transfer kinetics. When the formation of rGO/PANI composite, the aforementioned drawbacks of PANI could be overcome and also an aid to prevent the aggregation of rGO sheets through synergistic effects.

Besides, PANI acts as a bridge to facilitate the dispersion of mono or bimetallic nanoparticles over rGO sheets through electrostatic interactions, which could be an effective strategy for improving the stability of rGO/PANI/Pd or Pd: Au nanocomposites, surface area and catalytic active sites. Recently, Pd or Au and carbon-supported Pd or Au NPs have been reported as catalysts for the reduction of nitro compounds through Suzuki-Miyaura coupling reactions^35^. Likewise, Fe based Fischer-Tropsch catalysts, while Pt and GO supported Pt: Pd NPs were reported for methanol electro-oxidation^36^. Therefore, the research effort on bimetallic nanoparticles have been received significant attractive attention, owing to its high catalytic activity and better durability than that of mono metals. To the best of the author’s knowledge, there are no reports on the development of rGO-PANI supported mono (Pd) & bimetallic (Pd: Au) nanoparticles for the reduction of organic/inorganic pollutants and antimicrobial activities in waste-water. Hence, authors are motivated to prepare bare GO, rGO, PANI, three different ratios of rGO-PANI_(80:20, 50:50, 10:90)_ and rGO-PANI_(80:20)_/Pd & Pd:Au_(1:1, 1:2_, _2:1)_ nanocomposite catalysts by a simple chemical reduction method and analyze the impact of a combination of graphene, PANI and mono & bimetallic nanoparticles through different characterization techniques in order to find out their structure, morphology, electronic distribution, catalytic and antimicrobial activities.

## Experimental

### Chemicals

Graphite powder (Alfa Aesar), aniline (Alfa Aesar), sulphuric acid (SRL), phosphoric acid (Alfa Aesar), hydrochloric acid (SRL), ammonium peroxydisulfate (Alfa Aesar), potassium permanganate (Sigma-Aldrich), potassium tetrachloropalladate (Alfa Aesar), tetrachloro auric acid (Alfa Aesar), polyvinyl pyrrolidone (Alfa Aesar), sodium borohydride (Alfa Aesar), hydrazine (Sigma-Aldrich), hydrogen peroxide (SRL), ethanol (SRL), hexane (Alfa Aesar), double distilled water were used as a starting precursors for the preparation of bare rGO, PANI, and rGO-PANI supported mono (Pd) & bimetallic (Pd: Au) nanocomposite hybrid catalysts.

### Synthesis of reduced graphene oxide (rGO)

Graphite oxide (GO) was synthesized from graphite powder adopting an improved method^37^. Chemical conversion of GO into rGO was done according to the reported method^38^. In a typical experiment, 1 g of GO was dispersed in 500 mL of deionized water (DI). Then, 1 mL of hydrazine monohydrate was added, and the mixture was heated at 95 °C for 2 h. Once the reaction was completed, the rGO was collected by filtration as a black powder. The obtained rGO cake was washed with DI water several times to remove the unreacted hydrazine and dried in a vacuum oven at 80 °C for 24 h to get the final product.

### Synthesis of three different ratios of rGO-PANI_(80:20, 50:50, 10:90)_ composites

The three different ratios of rGO-PANI_(80:20, 50:50, 10:90)_ composites were synthesized by adopted the previously reported method^39^ with slight modifications. In a typical synthesis process, first, the required amount of aniline monomer was dissolved in 1 M HCl at a concentration of 0.3 M for 1 L. The 80 mg of as-prepared rGO was dispersed in DI water with the aid of ultrasonication bath for 1 h, subsequently, aniline was added (taken from 0.3 M for 20 ml) into the rGO dispersed solution under continuous stirring at room temperature. In addition to this, ammonium peroxydisulfate solution with a mole ratio to aniline of 1:4 in 1 M HCl was rapidly poured into the above mentioned resultant mixed solution under vigorous stirring at room temperature. After 5 min, the resultant mixture solution colour was changed into green, and then diluted by 100 mL of DI water by continuous stirring at room temperature. The resultant rGO-PANI_(80:20)_ composites were collected by filtration and repetitively washed with DI water, ethanol and hexane until the filtrate becomes colourless and dried in a vacuum oven at 80 °C for 24 h. The same procedure was adopted for the synthesis of other two composites [rGO-PANI_(50:50)_ - rGO-50 mg, aniline monomer taken from 0.3 M for 50 ml] and [rGO-PANI_(10:90)_ - rGO-10 mg, aniline monomer taken from 0.3 M for 90 ml] with different ratios of rGO and PANI.

### Synthesis of rGO-PANI_(80:20, 50:50, 10:90)_/Pd monometallic nanocomposite hybrid catalysts

In this synthesis processes, first, 0.05 mM of potassium tetrachloropalladate solution was taken in 100 ml round bottom (RB) flask contains 60 mg of PVP. The reaction mixture was stirred at 50 °C for 4 h. Then, the rGO-PANI_(80:20)_ composite solution (sonicated 5 mg for 2 h) was added under continuous stirring at 70 °C for 24 h. Subsequently, 0.4 M of sodium borohydride (NaBH_4_) solution was added to the reaction mixtures and allowed to continue stirring for 2 h. Finally, the resulted product was collected by centrifugation using DI water and ethanol for three times and kept in a hot air vacuum oven at 70 °C for 24 h to remove the solvents for further studies. Similarly, the other two different ratios of rGO-PANI_(50:50, 10:90)_ composites supported Pd monometallic hybrid catalysts were prepared by the same procedure for comparison.

### Synthesis of rGO-PANI_(80:20)_/Pd:Au_(1:1, 1:2, 2:1)_ bimetallic nanocomposite hybrid catalysts

**Figure 1:**
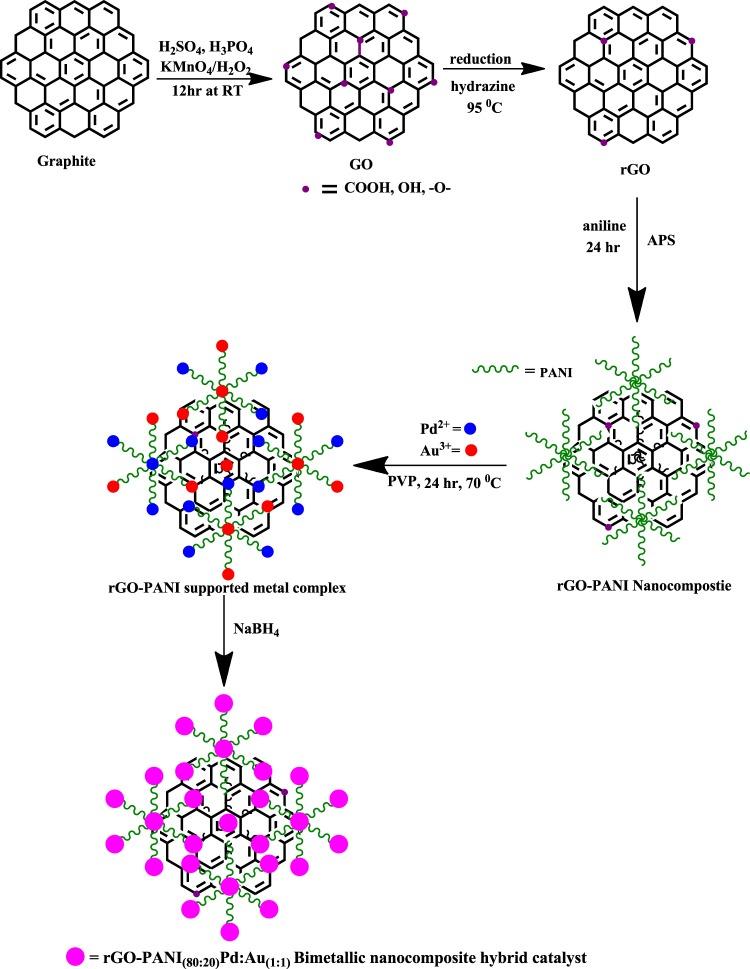
Pictorial representation for the preparation of rGO-PANI_(80:20)_/Pd:Au_(1:1)_ bimetallic nanocomposite hybrid catalyst.

In this process, 0.05 mM of potassium tetrachloropalladate and chloroauric acid were taken at a mole ratio of 1:1, in a 100 ml (RB) flask contained 60 mg of PVP. The reaction mixture was stirred at 50 °C for 4 h. Then, an optimized rGO-PANI_(80:20)_ composite solution (sonicated 5 mg for 2 h) was added to the above-mentioned mixtures under continuous stirring at 70 °C for 24 h. Subsequently, 0.4 M of sodium borohydride solution was added and allowed to continue stirring for 2 h. Finally, the resulted product was collected by centrifugation using DI water and ethanol for several times and dried in hot air vacuum oven at 70 °C for 12 h. Similar procedure has been followed for the preparations of bimetallic nanocomposite hybrid catalysts [rGO-PANI_(80:20)_/Pd:Au_(1:2)_ and rGO-PANI_(80:20)_/Pd:Au_(2:1)_] with the other two different ratios of Pd:Au bimetallic NPs for comparison. For better understanding, the complete processes for the formation of rGO-PANI_(80:20)_ supported Pd:Au_(1:1)_ bimetallic nanocomposite hybrid catalyst is depicted in the form of pictorial representation in Fig. 1.

## Results and Discussions

### Structural studies

**Figure 2:**
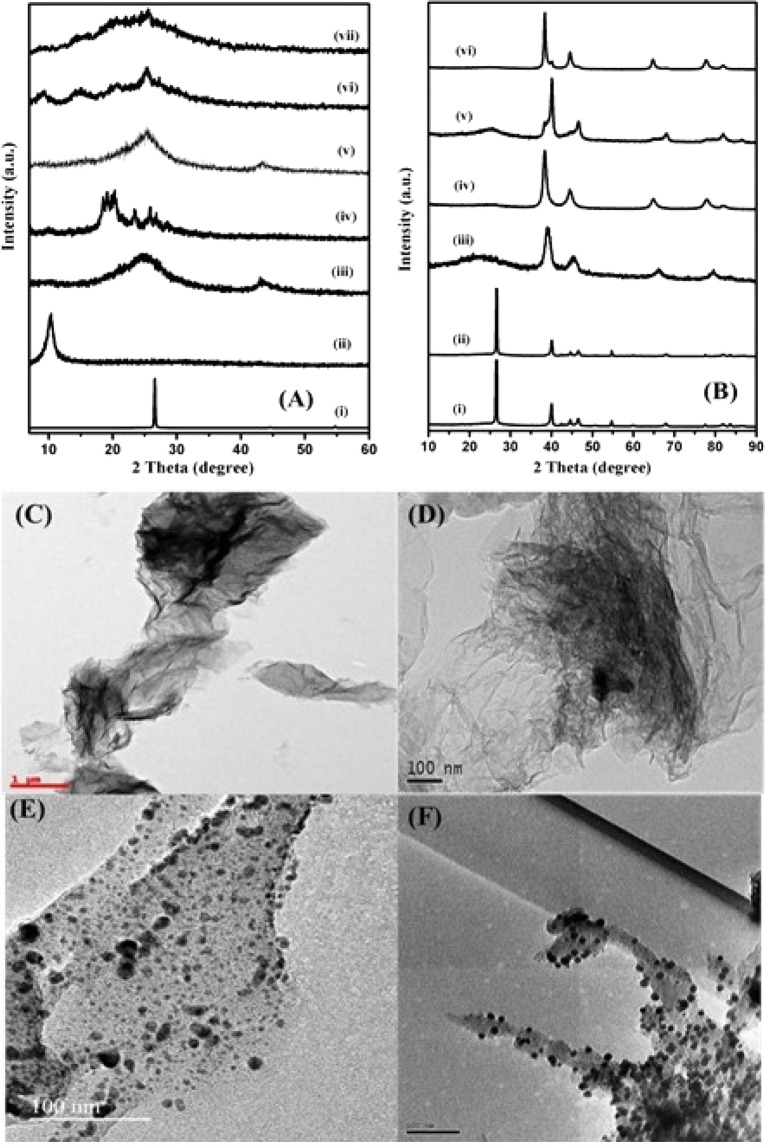
**(A)** XRD patterns of (i) graphite, (ii) GO, (iii) rGO, (iv) PANI, (v) rGO-PANI_(80:20)_, (vi) rGO-PANI_(50:50)_, (vii) rGO-PANI_(10:90)_; **(B)** XRD patterns of (i) rGO-PANI_(80:20)_/Pd NPs, (ii) rGO-PANI_(50:50)_/Pd NPs, (iii) rGO-PANI_(10:90)_/Pd NPs, (iv) rGO-PANI_(80:20)_/Pd:Au_(1:1)_, (v) rGO-PANI_(80:20)_/Pd:Au_(1:2)_, (vi) rGO-PANI_(80:20)_/Pd:Au_(2:1)_; HRTEM images of **(C)** rGO; **(D)** rGO-PANI_(80:20)_; **(E)** rGO-PANI_(80:20)_/Pd; **(F)** rGO-PANI_(80:20)_/Pd:Au_(1:1)_ bimetallic nanocomposite hybrid catalysts.

The X-ray diffraction (XRD) patterns of pure graphite, as-prepared GO, rGO, three different ratios of rGO-PANI_(80:20, 50:50, 90:10)_ composites and rGO-PANI_(80:20, 50:50, 10:90)_ supported mono (Pd) & bimetallic [Pd:Au_(1:1, 1:2, 2:1)_] nanocomposite hybrid catalysts are shown in Fig. 2A,B. From Fig. 2A(i), the observed X-ray diffraction pattern of pure graphite showed well defined high-intensity diffraction peak at 2θ = 26.7° corresponds to the (002) lattice planes with a d-spacing of 3.35 Å compared with the standard JCPDS data (Card No. 98-005-2916). Likewise, the observed diffraction peak of as-prepared GO was shifted towards the lower 2θ value of 10.27°, which specifies that the graphite was completely oxidized and confirms the formation of GO as shown in Fig. 2A(ii). Whereas, in the case of rGO, a new broad and low-intensity diffraction peak was observed at 2θ = 24.16°, which clearly depicts the formation of rGO through the chemical reduction method as shown in Fig. 2A(iii). In the XRD pattern of pure PANI, it was observed that amorphous nature as shown in Fig. 2A(iv). In the case of three different ratios of rGO-PANI_(80:20, 50:50, 10:90)_ composites, the intensity of rGO diffraction peak was decreased and broadened at a lower concentration as shown in Fig. 2A(v). It attributes the formation of a more disordered nature through an intercalation and exfoliation of PANI and rGO nanosheets, respectively. Further, the addition of PANI into rGO, the intensity of diffraction peak was slightly increased and observed the new diffractions peaks are shown in Fig. 2A(vi–vii). These results depict the formation of the PANI crystalline phase over the rGO nanosheets, which may lead to suppressing the catalytic & microbial activities.

In addition to this, the diffraction patterns of rGO-PANI supported mono (Pd) [rGO-PANI_(80:20, 50:50, 10:90)_/Pd] & bimetallic (Pd:Au) [rGO-PANI_(80:20_/Pd:Au_(1:1, 1:2, 2:1)_] nanocomposite hybrid catalysts were recorded and are shown in Fig. 2B(i-vi). The observed diffraction pattern of each sample showed both low and high-intensity diffraction peaks and are indexed with the corresponding lattice planes (111), (200), (220), (311) and (222) by comparing with the standard JCPDS data (Card No.: 46-1043, 04-0784) and confirmed the formation of nanocomposite hybrid catalysts with an uniform distribution of mono (Pd) & bi-metallic (Pd: Au) NPs over the rGO-PANI nanosheets^40–42^. Further, it was confirmed from the surface morphologies, elemental analysis and spectroscopy studies in the forthcoming sections.

In order to sight the surface morphologies, the as-prepared rGO, PANI, three different ratios of rGO-PANI_(80:20, 50:50, 10:90)_ composites, rGO-PANI_(80:20, 50:50, 10:90)_ supported monometallic (Pd) and an optimized rGO-PANI_(80:20)_ supported bimetallic [Pd: Au_(1:1, 1:2, 2:1)_] nanocomposite hybrid catalysts were investigated using SEM, FE-SEM and HR-TEM techniques are shown in Figs. (S1–S4) and 1C–1F. The HR-TEM image of as-prepared rGO showed the formation of ultrathin nanosheets with distinct curled edges and transparent lined surfaces as shown in Fig. 1C. When, the addition of PANI into rGO nanosheets, the morphology of rGO sheets were changed into a typical fibrillar structure as shown in Fig. 1D. In the same way, the HR-TEM images of an optimized rGO-PANI_(80:20)_ supported mono (Pd) & bimetallic [Pd: Au_(1:1)_] nanocomposite hybrid catalyst samples were clearly depicts the presence of mono (Pd) & bimetallic [Pd: Au_(1:1)_] NPs with a uniform distribution over the surfaces of an optimized rGO-PANI_(80:20)_ sheets through synergistic effect as shown in Fig. 1E,F. The observed both mono (Pd) & bimetallic (Pd: Au) NPs are in a spherical shape and their particle sizes are found to be ~30 nm and ~20 nm, respectively. Moreover, the captured energy dispersive X-ray (EDX) spectra of both mono (Pd) & bimetallic (Pd: Au) NPs decorated nanocomposite hybrid catalysts confirms the presence of Pd and Au elements and are shown in Figs. S5 and S6. In addition to this, elemental mapping was also conducted to characterize the uniform distributions of bimetallic (Pd: Au) NPs in an optimized rGO-PANI_(80:20)_ composite as shown in Fig. S7. The uniform color distribution confirms the presence of Pd, Au, C, N and O elements in an optimized rGO-PANI_(80:20)_ supported bimetallic Pd: Au_(1:1)_ nanocomposite hybrid catalysts through synergistic effect and thus, it could be a suitable catalyst material. Further, it was confirmed form the catalytic and microbial activity studies in the forthcoming sections.

**Figure 3:**
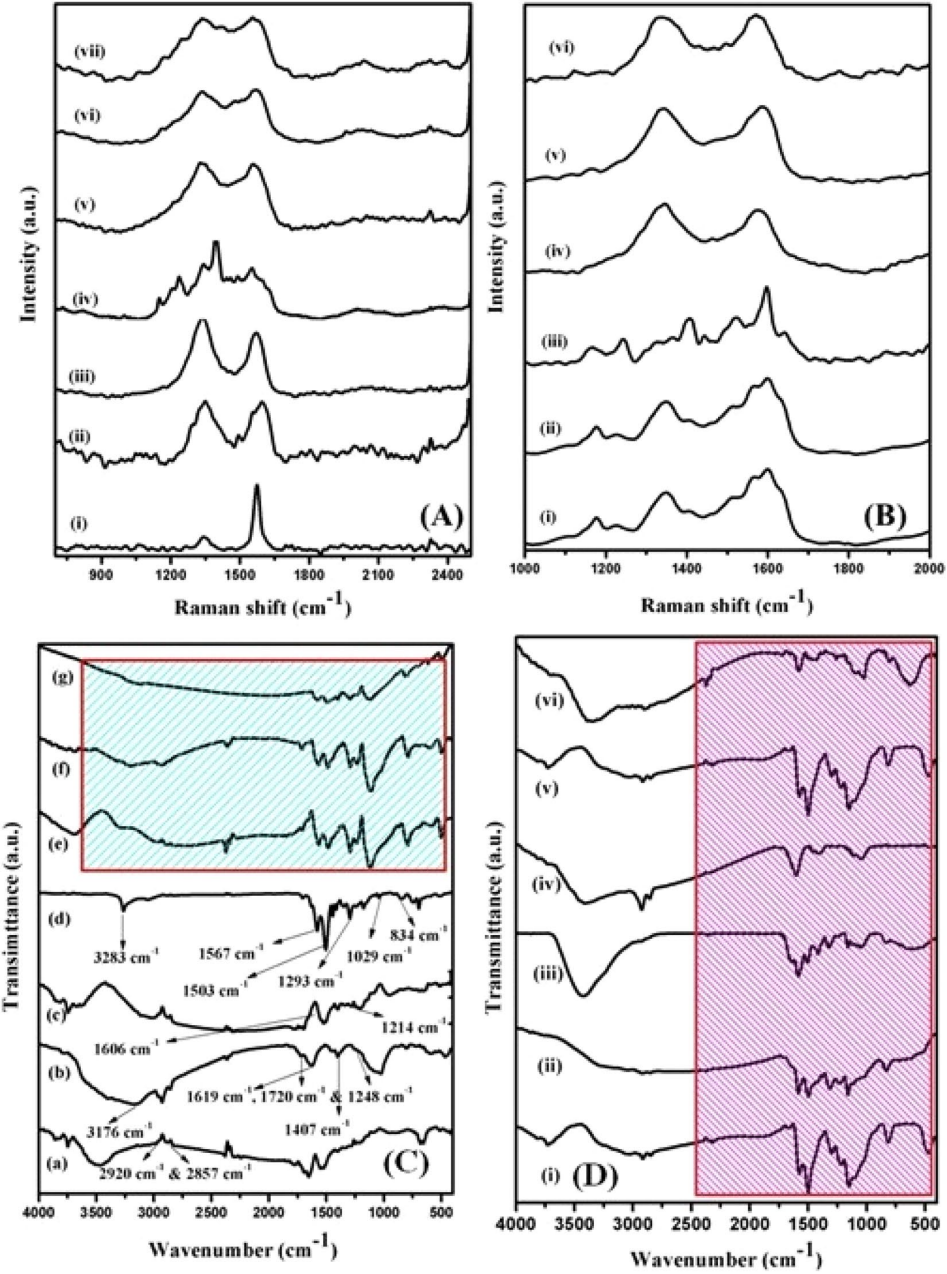
(**A)** Raman spectra of (i) graphite, (ii) GO, (iii) rGO, (iv) PANI, (v) rGO-PANI_(80:20)_, (vi) rGO-PANI_(50:50)_, (vii) rGO-PANI_(10:90)_ composites; **(B)** Raman spectra of (i) rGO- PANI_(80:20)_/Pd, (ii) rGO-PANI_(50:50)_/Pd, (iii) rGO-PANI_(10:90)_/Pd, (iv) rGO-PANI_(80:20)_/Pd:Au_(1:1)_, (v) rGO-PANI_(80:20)_/Pd:Au_(1:2)_, (vi) rGO-PANI_(80:20)_/Pd:Au_(2:1)_ nanocomposite hybrid catalysts; **(C)** FTIR spectra of (i) graphite, (ii) GO, (iii) rGO, (iv) PANI, (v) rGO-PANI_(80:20)_, (vi) rGO-PANI_(50:50)_, (vii) rGO-PANI_(10:90)_ composites; **(D)** FTIR spectra of (i) rGO-PANI_(80:20)_/Pd, (ii) rGO-PANI_(50:50)_/Pd, (iii) rGO-PANI_(10:90)_/Pd NPs, (iv) rGO-PANI_(80:20)_/Pd:Au_(1:1)_, (v) rGO-PANI_(80:20)_/Pd:Au_(1:2)_, (vi) rGO-PANI_(80:20)_/Pd:Au_(2:1)_ nanocomposite hybrid catalysts.

As we know that, Raman spectroscopy is a powerful technique to understand the structure of carbon-based materials, including graphene/graphite and carbon nanotubes (CNT), etc., by evaluating the intensity ratio (*I*_D_/*I*_G_) of both the characteristic D and G bands. The Raman spectra of bare graphite, as-prepared GO, rGO, PANI, three different ratios of rGO-PANI_(80:20, 50:50, 10:90)_ composites, and rGO-PANI_(80:20, 50:50, 10:90)_ supported mono (Pd) & bimetallic [Pd: Au_(1:1, 1:2, 2:1)_] nanocomposite hybrid catalysts with different ratios were shown in Fig. 3A,B. From Fig. 3A(i), the Raman spectrum of pure graphite showed two characteristic vibrational bands at 1318 cm^−1^ and 1579 cm^−1^ corresponds to the D and G bands, respectively. The intensity ratio (*I*_D_*/I*_G_) of D and G bands was calculated and is found to be ~ 0.2. When the graphite was oxidized into graphene oxide (GO), the intensity ratio (*I*_D_*/I*_G_) of D and G band was increased from ~0.2 to ~0.5, due to an intercalation of functional groups between the lattice planes, which lead to form the defects or disorder in the carbon structure as shown in Fig. 3A(ii). Further reduction of GO into rGO, the oxygen functional groups in the GO were removed, and the conjugated G network (sp^2^ carbon) will be re-established, and the size of the re-established G network is smaller than the original one, and thus, the intensity ratio (*I*_D_*/I*_G_) ratio of rGO is ~0.6, which is higher than GO as shown in Fig. 3A(iii). Thus, the experimental results in this study suggesting a successful reduction of GO into rGO. Furthermore, In Fig. 3A(iv), the Raman spectrum of bare PANI showed a characteristic vibrational bands at 1610, 1556, 1468, 1346, 1210 and 1180 cm^−1^, corresponds to the C–C stretching of the benzenoid ring, C=C stretching of the quinoid ring, C=N stretching of the quinoid ring, C–N stretching of the quinoid ring, C–N stretching of the benzenoid ring and C–H bending of the benzenoid ring, respectively. When, an increase in the concentrations of PANI_(20, 50, 90)_ into rGO_(80, 50, 10)_, each spectrum described the variations of both the characteristic D and G band intensity and also observed the minor peak shit towards the lower wavenumber are shown in Fig. 3A(v-vii). The intensity ratio of all the three different rGO-PANI_(80:20, 50:50, 10:90)_ composites were calculated and are found to be ~1, 0.9 & 0.8, respectively. These results confirm the formations of rGO-PANI composites through intermolecular interactions between rGO and PANI with respect to three different ratios of PANI contents. Moreover, the Raman spectra of rGO-PANI_(80:20, 50:50, 10:90)_ supported mono (Pd) & bimetallic [Pd:Au_(1:1, 1:2, 2:1)_] nanocomposite hybrid catalysts are illustrated in Fig. 3B(i-vi). It can be clearly seen in Fig. 3B(i-iii), the intensity of C=N stretching band of the quinoid ring was dramatically decreased and observed the new low-intensity bands in the mono (Pd) metallic nanocomposite hybrid catalysts. Besides, the intensity ratio (*I*_D_/*I*_G_) of all the three different rGO-PANI_(80:20, 50:50, 10:90)_ composites supported mono (Pd) metallic hybrid catalysts were calculated and are found to be higher (~1.7, 1.5 & 1.3, respectively) than that of bare rGO-PANI composites. Whereas, the nanocomposite hybrid catalysts with three different ratios of bimetallic [Pd: Au_(1:1, 1:2, 2:1)_] nanoparticles showed well-defined characteristic D and G bands with high-intensity ratio (~2.5, 1.9, 2.3, respectively) than that of mono (Pd) metallic as well as bare composite catalysts, which signifies the presence of more defects or disorder structure due to an exfoliation of rGO nanosheets. Also, it confirms the formation of nanocomposite hybrid catalysts through electrostatic interactions between rGO-PANI_(80:20)_ and Pd: Au_(1:1)_ NPs.

To further confirm the formation of surface complexes, the FTIR spectra of all the prepared bare GO, rGO, PANI, rGO-PANI_(80:20, 50:50, 10:90)_ and rGO-PANI supported mono (Pd) & bimetallic [Pd: Au_(1:1, 1:2, 2:1)_] nanocomposite hybrid catalyst samples were recorded and are illustrated in Fig. 3(C,D). As shown in Fig. 3C(i), the FTIR spectrum of pure graphite showed the characteristic vibrational bands at 2920 and 2857 cm^−1^ are attributed to the asymmetric and symmetric C–H stretching modes, respectively. In the case of GO, a strong vibrational band at 3176 and 1407 cm^−1^, corresponds to the O–H stretching vibration mode. Likewise, the bands at 1619, 1720 and 1248 cm^−1^ are attributed to the C–O (epoxy or alkoxy), C=O (carboxylic acid) and carbonyl moieties, respectively, as shown in Fig. 3C(ii). It clearly suggests that the graphite was completely oxidized by a chemical oxidative method. Whereas, after the reduction of GO into rGO using hydrazine, new absorption bands appeared at 1214 and 1606 cm^−1^ are attributed to the conjugated sp^2^ carbon atom as shown in Fig. 3C(iii). Besides, the FTIR spectrum of bare PANI displayed the transmittance bands at 1503 and 1567 cm^−1^ are correspond to the benzene and quinoid ring, respectively as shown in Fig. 3C(iv). Likewise, the bands appeared at 3283, 1293, 1029 and 834 cm^−1^ are attributed to the N-H, C-H stretching of the secondary aromatic amine, aromatic C-H in and out plane bending vibrations, respectively. When, the addition of three different ratios of PANI_(20, 50, 90)_ into rGO_(80, 50, 10)_, the each FTIR spectrum displayed both the characteristic vibrational bands in the measured region, which provides an evidence for the formation of surface complexes through an intermolecular interactions between the polymeric polaronic lattice of PANI and rGO nanosheets as shown in Fig. 3C(v–vii). Additionally, in the case of rGO-PANI supported mono (Pd) & bimetallic [Pd:Au] nanocomposite hybrid catalysts, the FTIR spectra exhibited a well-defined both the characteristic transmittance bands due to the synergistic effect between rGO-PANI composites and mono (Pd) & bimetallic (Pd:Au) nanoparticles, as a result, confirms the formation of hybrid catalysts are shown in Fig. 3D(i–vi).

**Figure 4:**
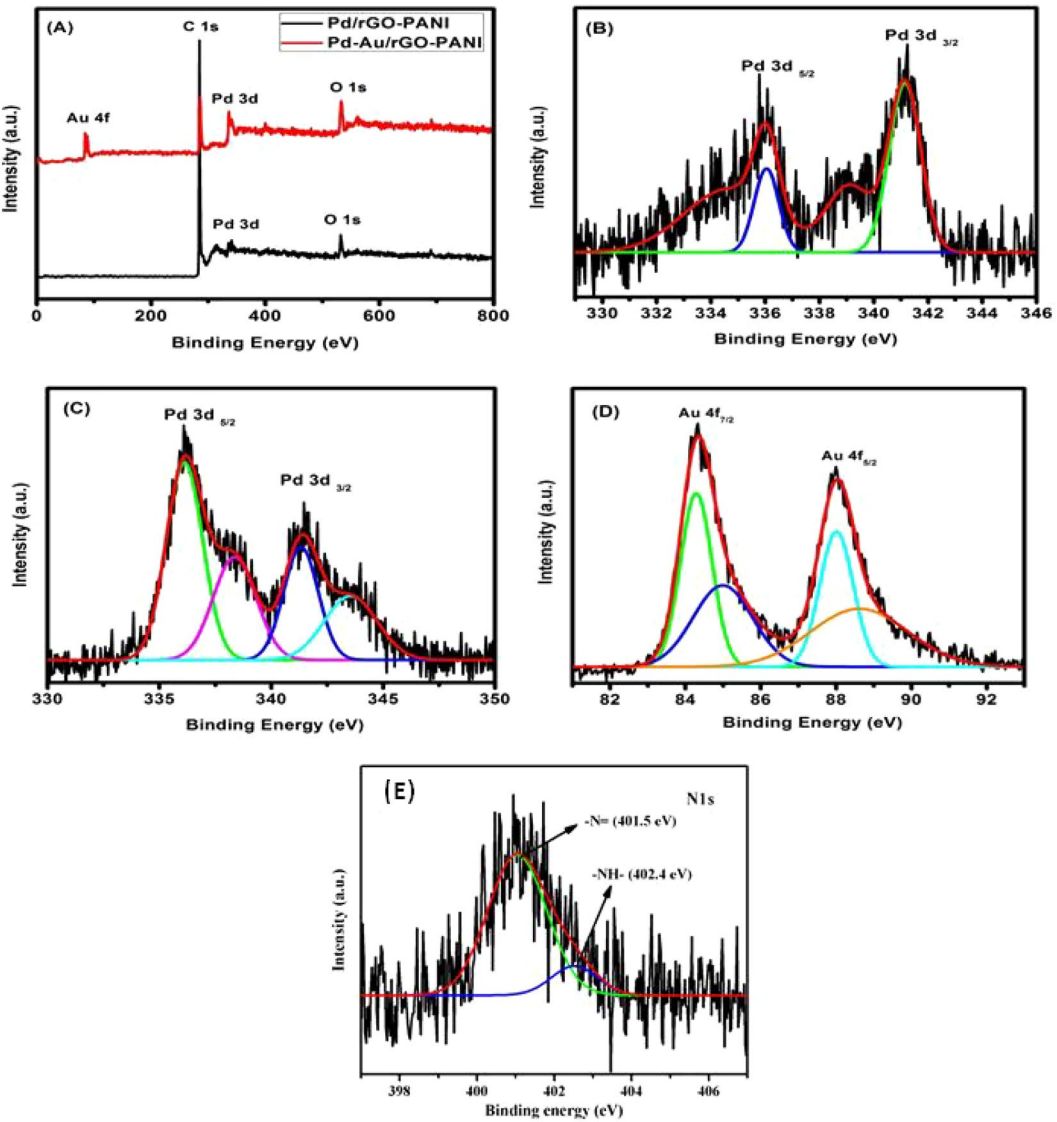
**(A)** XPS survey spectrum of an optimized rGO-PANI_(80:20)_/Pd and rGO-PANI_(80:20)_/Pd:Au_(1:1)_ nanocomposite hybrid catalysts; **(B)** Pd 3d spectrum of an optimized rGO- PANI_(80:20)_/Pd monometallic nanocomposite hybrid catalyst; **(C)** Pd 3d spectrum of an optimized rGO-PANI_(80:20)_/Pd:Au_(1:1)_ bimetallic nanocomposite hybrid catalyst; **(D)** Au 4f spectrum of an optimized rGO- PANI_(80:20)_/Pd:Au_(1:1)_ bimetallic nanocomposite hybrid catalyst; and **(E)** N1s spectrum of an optimized rGO-PANI_(80:20)_ supported bimetallic nanocomposite hybrid catalyst.

X-ray photoelectron spectroscopy (XPS) was used to characterize the electronic properties and chemical state information of as-prepared nanocomposite hybrid catalysts. Figure 4A showed the XPS spectrum of a full survey scan of an optimized rGO-PANI_(80:20)_ supported mono (Pd) & bimetallic [Pd: Au_(1:1)_] nanocomposite hybrid catalysts. From Fig. 4A, the spectrum displays Pd, Au, C and O elements. Further, revealing the presence of Pd state in an optimized rGO-PANI_(80:20)_ supported Pd monometallic hybrid catalyst in the high-resolution Pd 3d region, the binding energies of Pd 3d_5/2_ and Pd 3d_3/2_ is 335.8 eV and 340.9 eV, respectively, which confirmed the formation of Pd° metallic state as shown in Fig. 4B. In the case of bimetallic [Pd:Au_(1:1)_] NPs anchored hybrid catalysts, the binding energies of Pd 3d_5/2_ is 335.8 eV and Pd 3d_3/2_ 340.9 eV as well as Au 4f_7/2_ 84.3 eV and Au 4f_3/2_ 88.0 eV were confirmed the formation of Pd° and Au^0^ metallic state as shown in Fig. 4C,D^43,44^. Similarly, the binding energy values of N1s patterns for –N= and –NH– were evaluated and it was found to be 401.5 and 402.4 eV, respectively. This result suggests the occurrence of strong electronic interactions between metal nanoparticles [mono (Pd) & bimetallic (Pd: Au)] and rGO-PANI_(80:20)_ composite through synergistic effect owed to π-π stacking and van-der Waals force, which could effectively facilitate the betterment of catalytic activity towards the reduction of organic/inorganic pollutants.

### UV-Visible spectroscopy analysis

All the newly prepared bare and nanocomposites hybrid catalysts were characterized by UV-Visible spectroscopy (see Supporting Information Fig. S8A,B). In Fig. S8A(ii), the UV-visible spectrum of GO showed two absorption peaks at 225 and 299 nm, corresponds to the л-л* transition of aromatic C-C bond and C=O bond, respectively, which clearly illustrating the oxidation of graphite. In the case of rGO, the observed л-л* transition peak was shifted to 269 nm due to the complete reduction of GO into rGO as shown in Fig. S8A(iii). Similarly, Fig. S8A(iv) displays the UV-visible spectrum of pure PANI absorption peak at 276 nm, which attributes the transition of an electron from the highest occupied molecular orbital (HOMO) to lowest unoccupied molecular orbital (LUMO), (i.e., л-л* electronic transition). Furthermore, the absorption peaks at 387 and 565 nm corresponds to the polaron –л and -л* polar on benzenoid and quinoid excitonic transition, respectively. Fig. S8A(v–vii) shows the UV-visible spectra of rGO-PANI_(80:20, 50:50, 10:90)_ composites with different ratios. In these spectra, the rGO peak at 269 nm was shifted towards higher wavelength at 315 nm, 325 nm and 648 nm with respect to different rGO-PANI composites, which attributes the formation of rGO-PANI composites. In the case of rGO-PANI supported mono Pd nanocomposite hybrid catalysts, the UV-Vis spectra showed a broad peak with different features, which depicts the influences of mono Pd metal NPs interactions over the surface of three different rGO-PANI_(80:20, 50:50, 10:90)_ composites as shown in Fig. S8B(i–iii). Likewise, the UV-visible spectra of an optimized rGO-PANI_(80:20)_ supported three different ratios of bimetallic Pd:Au_(1:1, 1:2, 2:1)_ NPs showed the characteristic peak at 523 nm corresponds to Au metal as shown in Fig. S8B(iv–vi). At a higher ratio of Pd metal, the peak at 523 nm was disappeared. All these results suggested the formation of bimetallic Pd:Au nanocomposite hybrid catalysts through synergistic effect between bimetallic NPs and rGO-PANI_(80:20)_ composites. Furthermore, the catalytic and antimicrobial studies of all the newly prepared samples were demonstrated and it was described in the forthcoming sections.

### Catalytic activity studies

#### Catalytic reduction of p-NP in the presence of three different ratios of rGO-PANI_(80:20, 50:50, 10:90)_ supported Pd monometallic nanocomposite hybrid catalysts

The newly prepared bare GO, rGO, PANI, three different ratios of rGO-PANI_(80:20, 50:50, 10:90)_ composites and rGO-PANI_(80:20, 50:50, 10:90)_ supported Pd monometallic nanocomposite hybrid catalysts were used to evaluate and/or optimize the best catalytic reduction of p-Nitrophenol (p-NP) in the presence of sodium borohydride (NaBH_4_) as a reducing agent. The catalytic reduction was followed by pseudo-first-order reaction kinetics, and the reduction product was analyzed by UV-Visible spectrophotometer at a regular interval of time. Moreover, time-dependent UV-Vis absorption spectra of p-nitrophenol (p-NP) reduction processes were demonstrated using an optimized rGO-PANI_(80:20)_ supported Pd monometallic nanocomposite hybrid catalyst (see Supplementary Information Fig. S9). The prominent absorbance peak of p-NP reduction was observed at 400 nm in the absence of a catalyst. When the addition of an optimized rGO-PANI_(80:20)_ supported Pd monometallic hybrid catalyst into the reaction system, the absorbance peak at 400 nm decreases and at the same time a new absorption peak at 300 nm gradually increases with respect to time. For comparison, the same experimental procedure was followed for the reduction of p-NP using bare GO, rGO, three different ratios of bare rGO-PANI_(80:20, 50:50, 10:90_ and rGO-PANI_(80:20, 50:50, 10:90)_ supported Pd monometallic nanocomposite hybrid catalysts. The rate constants for the reduction of p-nitrophenol using all the newly prepared bare and nanocomposite hybrid catalysts were calculated by follows the pseudo-first-order reaction kinetics, and the obtained results are summarized in Table 1. As can be seen in Table 1, the ratio of bare rGO-PANI_(80:20)_ and monometallic (Pd) anchored rGO-PANI_(80:20)_ nanocomposite hybrid catalysts exhibit higher catalytic activity than that of other composites. This might be due to high catalytic active sites through better synergistic effect and thus, it may lead to enhance the excellent photocatalytic activity owing to the electron transfer from the rGO-PANI to Pd metal NPs. Moreover, it also evidences for the formation of surface complexes between rGO-PANI_(80:20)_ and Pd metal NPs as proven by Raman and FT-IR spectra. Hence, the rGO-PANI_(80:20)_ composite ratio was chosen as the right support for the preparation of nanocomposite hybrid catalysts with three different ratios of bimetallic Pd: Au_(1:1, 1:2, 2:1)_ nanoparticles for further studies.

**Table 1:** Reduction of p-Nitrophenol in the presence of bare GO, rGO, PANI, three different ratios of rGO-PANI_(80:20, 50:50, 10:90)_ and PANI_(80:20, 50:50, 10:90)_ supported monometallic (Pd) nanocomposite hybrid catalysts.

S. No	Nanocatalysts	*k*_*obs*_ × 10^−3^ S^−1^
1	GO	0.7
2	rGO	1.2
3	PANI	0.5
**4**	**rGO-PANI**_**(80:20)**_	**3.2**
5	rGO-PANI_(50:50)_	2.7
6	rGO-PANI_(10:90)_	2.4
**7**	**rGO-PANI**_**(80:20)**_**Pd**	**4.7**
8	rGO-PANI_(50:50)_Pd	4.2
9	rGO-PANI_(10:90)_Pd	3.9

#### Catalytic reduction of Nitroaromatics using an optimized rGO-PANI_(80:20)_ supported Pd:Au_(1:1, 1:2, 2:1)_ bimetallic nanocomposite hybrid catalysts

**Figure 5:**
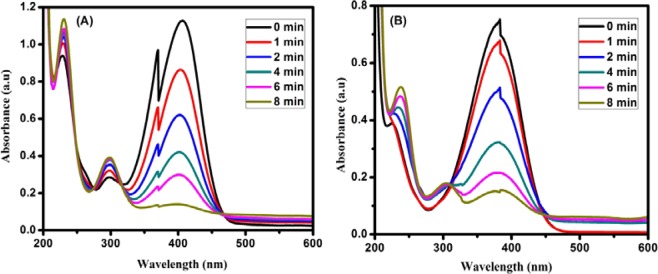
Time-dependent UV–Vis spectra of **(A)** p-Nitrophenol and **(B)** p-Nitroaniline reduction using NaBH_4_ as a reducing agent in the presence of an optimized rGO-PANI_(80:20)_ supported Pd: Au_(1:1)_ bimetallic nanocomposite hybrid catalyst.

**Figure 6:**
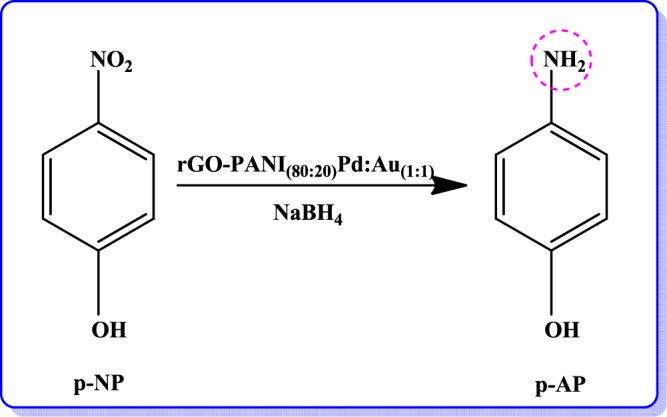
Reduction of p-Nitrophenol in the presence of an optimized rGO-PANI_(80:20)_ supported Pd:Au_(1:1)_ bimetallic nanocomposite hybrid catalyst.

**Figure 7:**
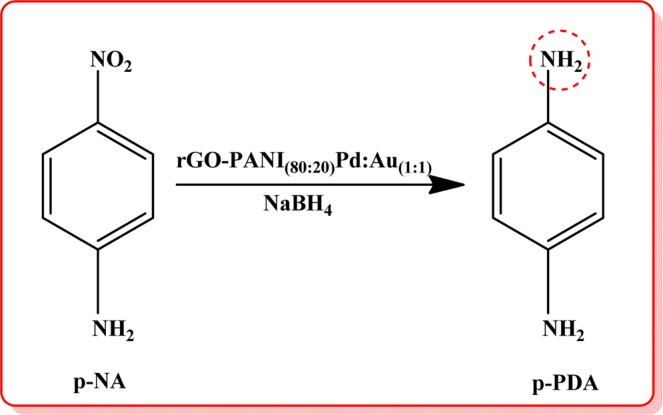
Reduction of p-Nitroaniline in the presence of an optimized rGO-PANI_(80:20)_ supported Pd:Au_(1:1)_ bimetallic nanocomposite hybrid catalyst.

In order the study the effect of three different ratio of bimetallic Pd:Au_(1:1, 1:2, 2:1)_ NPs anchored over an optimized rGO-PANI_(80:20)_ nanocomposite hybrid catalysts, the catalytic reduction processes of p-nitrophenol (p-NP) and p-nitroaniline (p-NA) were analysed by UV-Visible spectrophotometer in the presence of NaBH_4_ at fixed intervals of time, and the recorded spectra are illustrated in Fig. 5(A,B). The catalytic reduction was followed by pseudo-first-order reaction kinetics. As can be seen in Fig. 5(A,B), the absorbance peak observed at 400 nm for p-NP and 385 nm for p-NA decreased and also the other peaks at 300 nm and 238 nm are gradually increased, corresponding to p-aminophenol and p-phenylenediamine, respectively. These results confirm the complete reduction of p-NP, as well as p-NA in the presence of rGO-PANI(80:20), supported Pd:Au_(1:1)_ bimetallic nanocomposite hybrid catalyst. The overall rate constant values were calculated and the results are given in Tables 2 and 3. As can be seen from Tables 2 and 3, the rate constant values of nanocomposite hybrid catalysts were varied with respect to the different ratio of bimetallic Pd:Au_(1:1, 1:2, 2:1)_ nanoparticles, due to the variation of surface active sites and number of electrons transfer between Pd:Au_(1:1, 1:2, 2:1)_ nanoparticles and rGO-PANI_(80:20)_ nanosheets. However, the nanocomposite hybrid catalyst with the presence of Pd:Au_(1:1)_ nanoparticles ratio exhibited higher catalytic activity than other bimetallic composites. After the completion of catalytic reduction, the used bimetallic nanocomposite hybrid catalyst was recovered, and it was reused for five successive cycles for the reduction of nitro organics (p-NP and p-NA) in order to evaluate the stability or reusable of an optimized rGO-PANI_(80:20)_ supported Pd:Au_(1:1)_ bimetallic nanocomposite hybrid catalyst. In each cycle, the rate constant (*k*_obs_) values for both the nitro organics (p-NP and p-NA) are slightly reduced, and the results are given in Tables 4 and 5. Moreover, the reduction process of p-NP and p-NA in the presence of an optimized rGO-PANI_(80:20)_ supported Pd: Au_(1:1)_ bimetallic nanocomposite hybrid catalyst is depicted in Figs. 6 and 7.

**Table 2:** Rate constant values for the reduction of p-NP in the presence of an optimized rGO-PANI_(80:20)_ supported Pd: Au_(1:1, 1:2, 2:1)_ bimetallic nanocomposite hybrid catalysts.

S. No	Nanocatalysts	*k*_obs_ × 10^−3^ S^−1^
1	rGO-Pd	4.4
2	rGO/Pd:Au_(1:1)_	5.2
**3**	**rGO-PANI**_**(80:20)**_**/Pd:Au**_**(1:1)**_	**5.8**
4	rGO-PANI_(80:20)_/Pd:Au_(1:2)_	5.0
5	rGO-PANI_(80:20)_/Pd:Au_(2:1)_	5.5

**Table 3:** Rate constant values for the reduction of p-NA in the presence of an optimized rGO-PANI_(80:20)_ supported Pd: Au_(1:1, 1:2, 2:1)_ bimetallic nanocomposite hybrid catalysts.

S. No	Nanocatalysts	*k*_obs_ × 10^−3^ S^−1^
1	rGO-Pd	4
2	rGO/Pd:Au_(1:1)_	4.8
**3**	**rGO-PANI**_**(80:20)**_**/Pd:Au**_**(1:1)**_	**5.4**
4	rGO-PANI_(80:20)_/Pd:Au_(1:2)_	4.6
5	rGO-PANI_(80:20)_/Pd:Au_(2:1)_	5.0

**Table 4:** Reusable for the reduction of p-NP in the presence of an optimized rGO-PANI_(80:20)_ supported Pd:Au_(1:1)_ bimetallic nanocomposite hybrid catalyst.

S. No	Nanocatalysts	*k*_obs_ × 10^−3^ S^−1^
**1**	**rGO-PANI**_**(80:20)**_**/Pd:Au**_**(1:1)**_	**5.8**
2	1^st^ cycle	5.6
3	2^nd^ cycle	5.5
4	3^rd^ cycle	5.3
5	4^th^ cycle	5.1
6	5^th^ cycle	5.0

**Table 5:** Reusable for the reduction of p-NA in the presence of an optimized rGO-PANI_(80:20)_ supported Pd:Au_(1:1)_ bimetallic nanocomposite hybrid catalysts.

S. No	Nanocatalysts	*k*_obs_ × 10^−3^ S^−1^
**1**	**rGO-PANI**_**(80:20)**_**/Pd:Au**_**(1:1)**_	**5.4**
2	1^st^ cycle	5.3
3	2^nd^ cycle	5.1
4	3^rd^ cycle	5.0
5	4^th^ cycle	4.8
6	5^th^ cycle	4.6

#### Reduction of organic dyes

**Figure 8:**
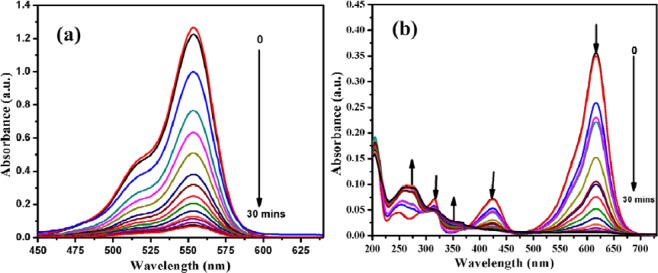
(**a)** Reduction of Rhodamine b (Rho b); **(b)** Reduction of Malachite green (MG) using NaBH_4_ as a reducing agent and rGO-PANI_(80:20)_/Pd:Au_(1:1)_ bimetallic nanocomposite hybrid catalyst.

The catalytic activity for the reduction of organic dyes such as Rhodamine b (Rho b) and Malachite green (MG) was examined in the presence of an optimized rGO-PANI_(80:20)_/Pd:Au_(1:1)_ bimetallic nanocomposite hybrid catalysts. The typical results of the time-dependent UV-Visible spectra of the reduction of an aqueous solution containing Rho b and MG dyes are illustrated in Fig. 8a,b, respectively. The maximum absorption peaks at 553 nm for Rho b and 617 nm for MG were observed in the absence of a catalyst. When the addition of 2 mg of rGO-PANI_(80:20)_/Pd: Au_(1:1)_ composite hybrid catalyst, the absorbance of both the dyes are decreased with the increase in time up to 30 min, and then, the reduction reaction attained a steady-state for both the dyes are shown in Fig. 8a,b. The overall pseudo-first-order rate coefficients values were calculated for both the Rho b and MG dyes, and the values are given in Tables 6 and 7.

**Table 6:** Rate constant values for the reduction of Rho b in the presence of an optimized rGO-PANI_(80:20)_ supported Pd:Au_(1:1, 1:2, 2:1)_ bimetallic nanocomposite hybrid catalysts.

S. No	Nanocatalysts	*k*_obs_ × 10^−3^ S^−1^
**1**	**rGO-PANI**_**(80:20)**_**/Pd:Au**_**(1:1)**_	**7.3**
2	rGO-PANI_(80:20)_Pd:Au_(1:2)_	5.7
3	rGO-PANI_(80:20)_Pd:Au_(2:1)_	6.2

**Table 7:** Rate constant values for the reduction of MG in the presence of an optimized rGO-PANI_(80:20)_ supported Pd:Au_(1:1, 1:2, 2:1)_ bimetallic nanocomposite hybrid catalysts.

S. No	Nanocatalysts	*k*_*obs*_ × 10^−3^ S^−1^
**1**	**rGO-PANI**_**(80:20)**_**/Pd:Au**_**(1:1)**_	**9.5**
2	rGO-PANI_(80:20)_/Pd:Au_(1:2)_	8.2
3	rGO-PANI_(80:20)_/Pd:Au_(2:1)_	8.4

**Figure 9:**
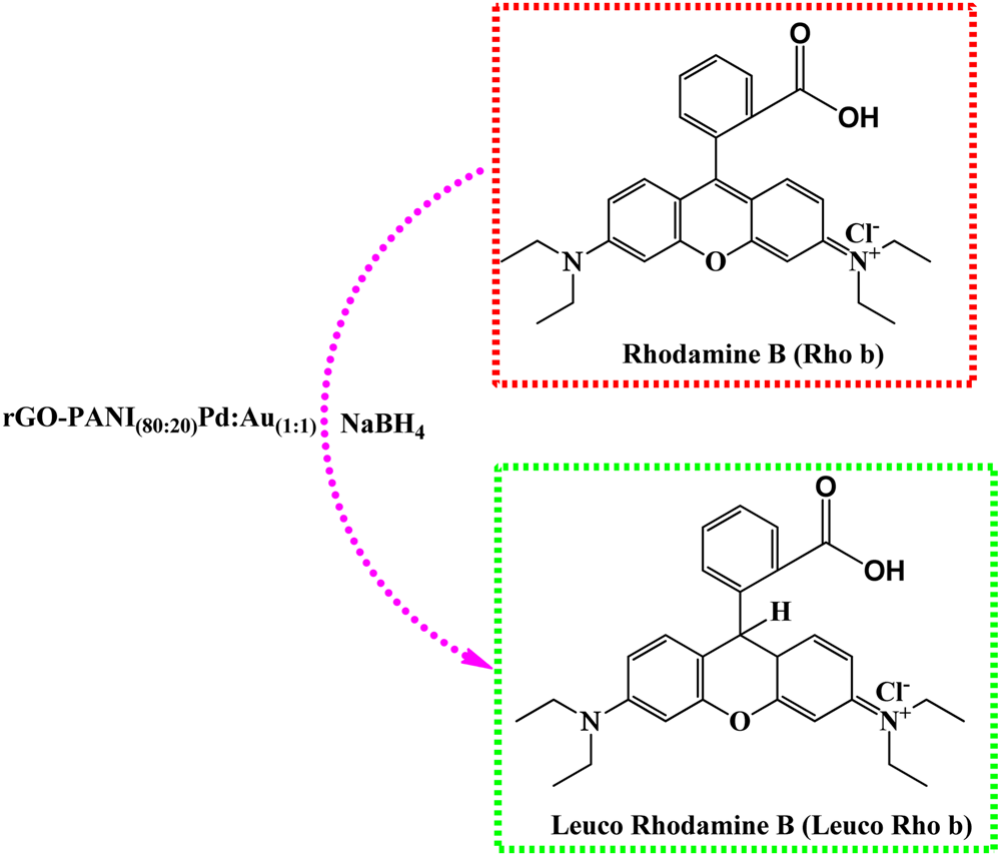
Reduction of Rhodamine B in the presence of an optimized rGO-PANI_(80:20)_ supported Pd:Au_(1:1)_ bimetallic nanocomposite hybrid catalyst.

**Figure 10:**
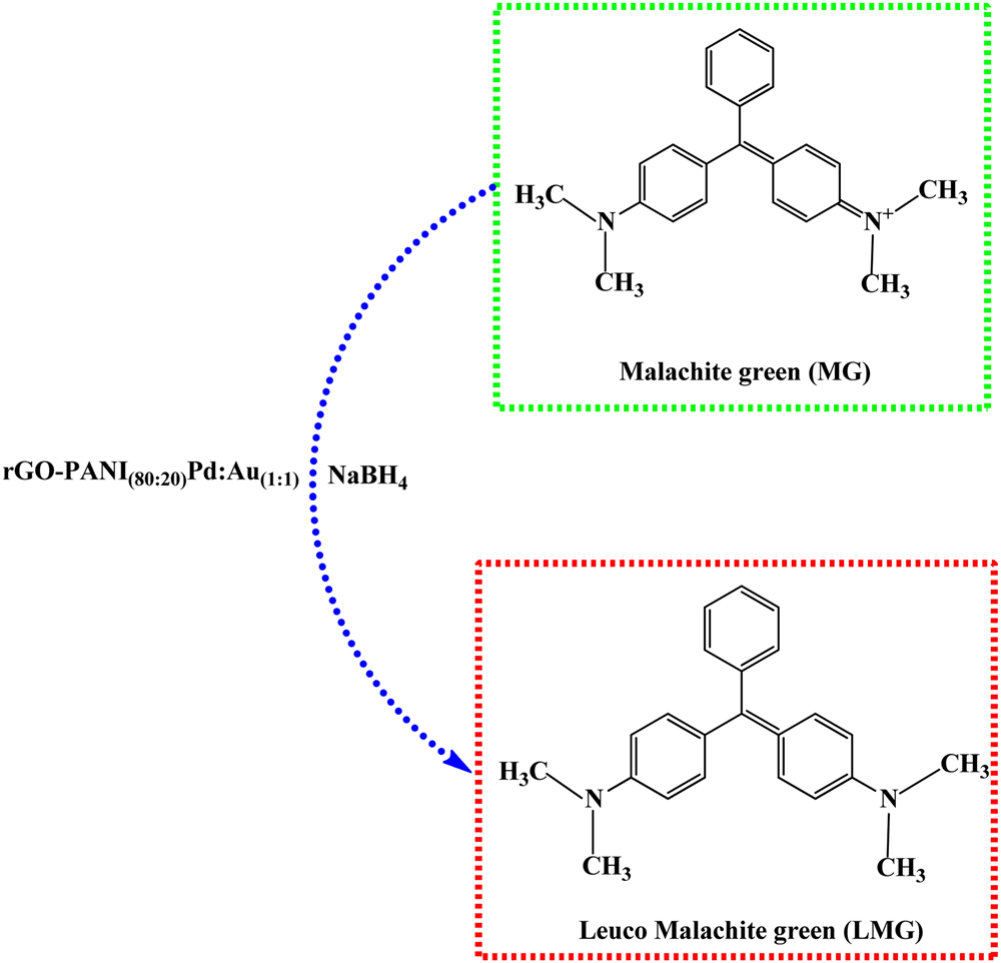
Reduction of Malachite green in the presence of an optimized rGO-PANI_(80:20)_ supported Pd: Au_(1:1)_ bimetallic nanocomposite hybrid catalyst.

Interestingly, an optimized rGO-PANI_(80:20)_/Pd: Au_(1:1)_ bimetallic nanocomposite hybrid catalyst exhibited superior catalytic activity than other bimetallic catalysts, which may be due to the presence of high catalytic active sites with better synergistic effect between rGO-PANI and bimetallic Pd:Au_(1:1)_ NPs. Once the reduction was completed, the used rGO-PANI_(80:20)_/Pd:Au_(1:1)_ bimetallic composite hybrid catalyst was collected and reused for five consecutive cycles for the reduction of both the Rho b and Malachite green (MG) dyes. In the end, the values of the rate constant (*k*_obs_) for both the dyes (Rho b and MG) were slightly reduced and are summarized in Tables 8 and 9. The reduction process of Rho b and Malachite green (MG) using rGO-PANI_(80:20)_ supported Pd: Au_(1:1)_ bimetallic composite hybrid catalyst is depicted in Figs. 9 and 10. It is well known that sodium borohydride (NaBH_4_) acts as a reducing agent in this typical reduction reaction. It rapidly decomposes on the sample of Pd: Au_(1:1)_ bimetallic nanoparticles yielding H^−^ ions. The H^-^ ions are retained on the surface of the bimetallic nanoparticles. They are then used to reduce either rhodamine b and malachite green to their colorless product (leuco base) as shown in the following reaction Figs. 9 and 10.

**Table 8:** Reusable for the reduction of Rhodamine b in the presence of an optimized rGO-PANI_(80:20)_ supported Pd:Au_(1:1)_ bimetallic nanocomposite hybrid catalyst.

S. No	Nanocatalysts	*k*_obs_ × 10^−3^ S^−1^
**1**	**rGO-PANI**_**(80:20)**_**/Pd:Au**_**(1:1)**_	**7.3**
2	1^st^ cycle	7.2
3	2^nd^ cycle	7.0
4	3^rd^ cycle	6.9
5	4^th^ cycle	6.7
6	5^th^ cycle	6.5

**Table 9:** Reusable for the reduction of Malachite green in the presence of an optimized rGO-PANI_(80:20)_ supported Pd:Au_(1:1)_ bimetallic nanocomposite hybrid catalyst.

S. No	Nanocatalysts	*k*_obs_ × 10^−3^ S^−1^
**1**	**rGO-PANI**_**(80:20)**_**/Pd:Au**_**(1:1)**_	**9.5**
2	1^st^ cycle	9.3
3	2^nd^ cycle	9.2
4	3^rd^ cycle	9.0
5	4^th^ cycle	9.3
6	5^th^ cycle	9.0

#### Removal efficiency of toxic metal ion

**Figure 11:**
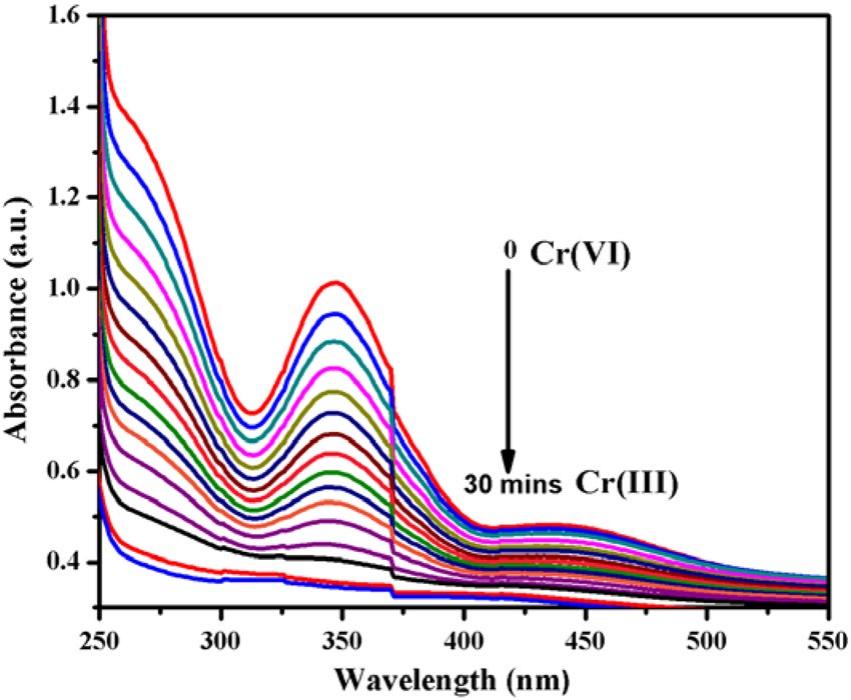
Time-dependent UV-vis spectra of reductive conversion of Cr(VI) to Cr(III) using formic acid (HCOOH) as a reducing agent in the presence of an optimized rGO-PANI_(80:20)_ supported Pd:Au_(1:1)_ bimetallic nanocomposite hybrid catalyst. Reaction conditions: [K_2_Cr_2_O_7_] = 20 mM, HCOOH = 1 ml, and catalyst amount = 2 mg (rGO-PANI_(80:20)_ supported Pd:Au_(1:1)_ bimetallic nanocomposite hybrid catalyst).

**Figure 12:**
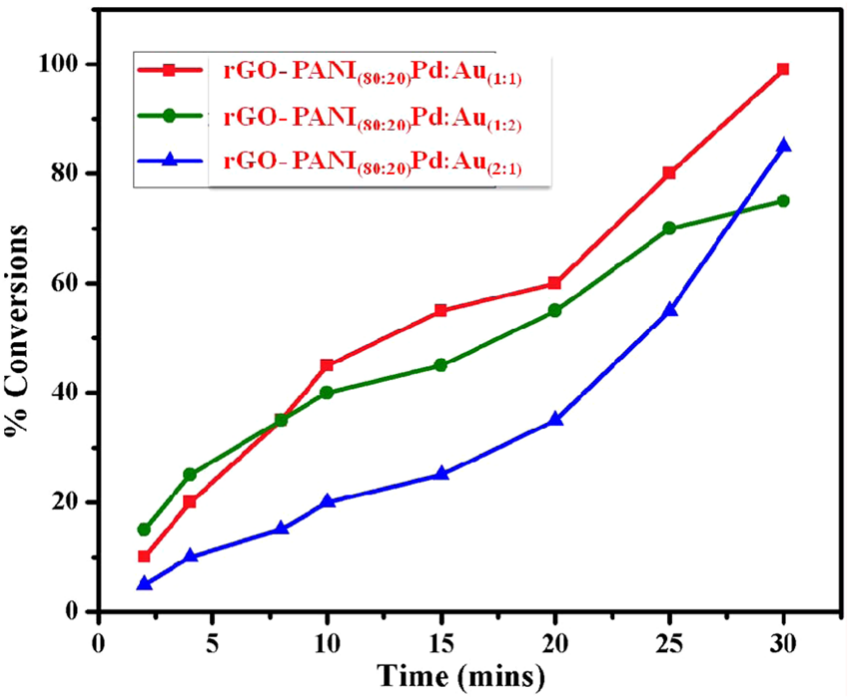
Overall catalytic reductive conversion of Cr(VI) to Cr(III) using an optimized rGO-PANI_(80:20)_ supported Pd:Au_(1:1, 1:2, 2:1)_ bimetallic nanocomposite hybrid catalysts.

**Figure 13:**
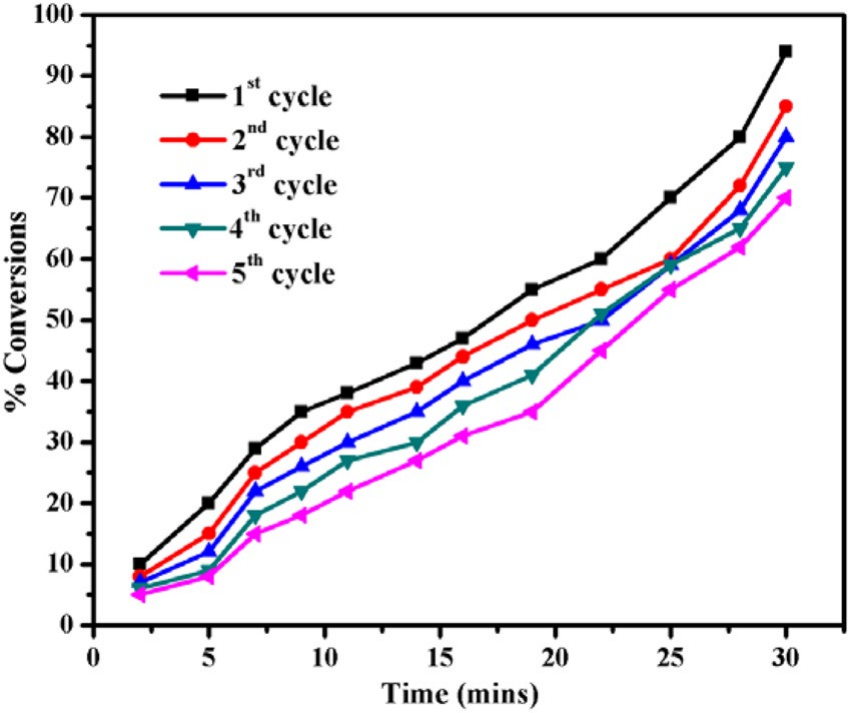
Recyclability of an optimized rGO-PANI_(80:20)_ supported Pd:Au_(1:1)_ bimetallic nanocomposite hybrid catalyst for their reductive conversion of Cr(VI) to Cr(III).

**Figure 14:**
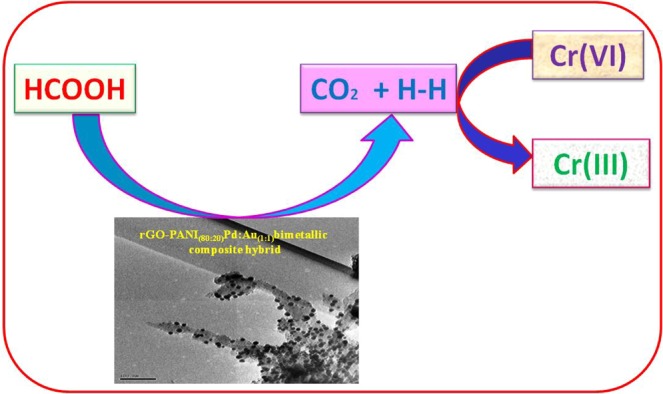
Reductive conversion of Cr(VI) to Cr(III) using HCOOH in the presence of an optimized rGO-PANI_(80:20)_ supported Pd:Au_(1:1)_ bimetallic nanocomposite hybrid catalyst.

Figure 11 shows the UV-visible spectra recorded at different time intervals for the reductive conversion of Cr(VI) to Cr(III) using an optimized rGO-PANI_(80:20)_/Pd: Au_(1:1)_ nanocomposite hybrid catalyst in the presence of formic acid as a reducing agent. In Fig. 11, the spectra showed a strong maximum absorbance peak at 350 nm, corresponds to Cr(VI). When, the addition of catalyst and reducing agent, the intensity of the maximum absorption peak gradually decreases with an increase in time and completely vanishes after 30 min, which indicates the complete reduction of Cr(VI) to Cr(III). At the end of the reaction, the colour of the reaction solution was changed from yellow to colourless. The formation of the product Cr(III) is confirmed by the excess addition of NaOH solution, which yielded a green colour solution due to the formation of hydroxy chromate (III) complex^45,46^. When the same reduction was carried out in the presence of formic acid without catalyst, no changes in colour of the solution. This result suggests that the reductive conversion of Cr(VI) is catalyst dependent. Hence, the simultaneous presence of HCOOH, as well as the catalyst in the reaction medium, is a prerequisite for the reduction of Cr(VI) to Cr(III). It is to be pointed out that the catalysts rGO-PANI_(80:20)_/Pd: Au_(1:2)_ and rGO-PANI_(80:20)_/Pd:Au_(2:1)_ exhibited much less activity than rGO-PANI_(80:20)_/Pd:Au_(1:1)_ nanocomposite hybrid catalyst for the same reductive conversion of Cr(VI) to Cr(III). The percentage of reductive conversion as a function of time was also calculated for all the bimetallic NPs, and the results are compared in Fig. 12. The activity of the catalysts was in the order of rGO-PANI_(80:20)_/Pd:Au_(1:1)_ > rGO-PANI_(80:20)_/Pd:Au_(2:1)_ > rGO-PANI_(80:20)_/Pd:Au_(1:2)_ nanocomposite hybrid catalysts. The catalytic activity of the rGO-PANI_(80:20)_/Pd:Au_(1:1)_ hybrid catalyst remained nearly same even after five successive cycles are shown in Fig. 13, and it confirms the stability and recyclability for the reduction of Cr(VI) to Cr(III). Hence, the reductive conversion process of Cr(VI) to Cr(III) in the presence of an optimized rGO-PANI_(80:20)_ supported Pd:Au_(1:1)_ bimetallic nanocomposite hybrid catalyst is depicted in Fig. 14.

### Antibacterial activity studies

**Figure 15:**
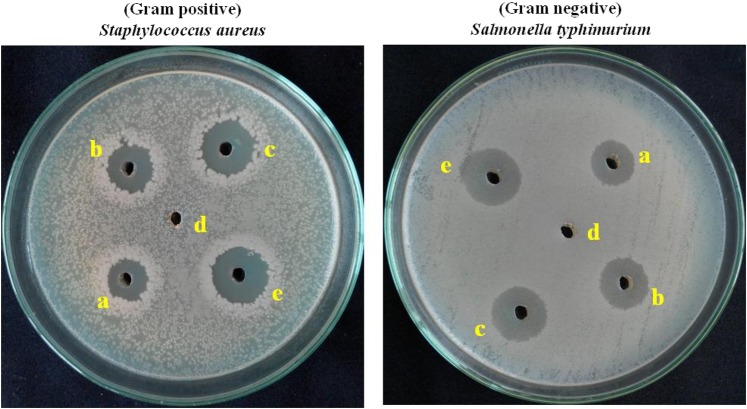
Antibacterial activity of an optimized rGO-PANI_(80:20)_ supported Pd:Au_(1:1)_ bimetallic nanocomposite hybrid catalyst against gram positive and gram-negative bacteria; (**a**) 50 μg/ml (**b**) 100 μg/ml (**c**) 150 μg/ml (**d**) DMSO and (**e**) Streptomycin as a standard drug.

The *in vitro* antibacterial activity of the catalysts with different ratios of Pd:Au_(1:1, 1:2, 2:1)_ was determined *by* the well diffusion method using human pathogens such as Gram-positive *Staphylococcus aureus* (*S. aureus*) and Gram-negative *Salmonella typhimurium* (*S. Typhi*) with bacterial cell cultures. The drug Streptomycin was used as a positive control drug in the study of antibacterial activity. All the three different ratios of bimetallic Pd: Au_(1:1, 1:2, 2:1)_ nanoparticles anchored over an optimized rGO-PANI_(80:20)_ hybrid catalysts were used with different dosages (50, 100 and 150 µg/mL) during the study. The antibacterial activity of rGO-PANI_(80:20)_ supported Pd: Au_(1:1)_ hybrid catalyst was measured against both the bacteria as shown in Fig. 15. The activities of the catalysts were determined by measuring the zone of inhibition for each dosage against each bacteria, and the results are presented in Tables 10 and 11. It is inferred that an optimized rGO-PANI_(80:20)_/Pd: Au_(1:1)_ bimetallic nanocomposite catalyst exhibited higher antibacterial activity than other catalysts, and also the zone of inhibition was almost close to that of streptomycin. The higher activity of this catalyst is ascribed to the small size of the bimetallic nanoparticles compared to the other catalysts. We hope that the rGO-PANI_(80:20)_/Pd:Au_(1:1)_ hybrid catalyst could easily bind to an amine or sulphur sites of the bacterial enzymes and inactivate them. So, the food material may not be degraded and the bacteria may not multiply. It was also observed the zone of inhibition with increasing the dosage of each catalyst.

**Table 10:** Antibacterial activity of an optimized rGO-PANI_(80:20)_ supported Pd:Au_(1:1, 1:2, 2:1)_ bimetallic nanocomposite hybrid catalyst against *Staphylococcus aureus*.

Organism	Catalysts	Concentration of catalyst (μg/ml)				
		50		100		150
		Zone of inhibition (mm)				
*Staphylococcus aureus*	**rGO-PANI**_**(80:20)**_**/Pd:Au**_**(1:1)**_	**11**	**13**		**15**	
	rGO-PANI_(80:20)_/Pd:Au_(1:2)_	7	10		11	
	rGO-PANI_(80:20)_/Pd:Au_(2:1)_	9	10		12	
	Streptomycin		17

**Table 11:** Antibacterial activity of an optimized rGO-PANI_(80:20)_ supported Pd:Au_(1:1, 1:2, 2:1)_ bimetallic nanocomposite hybrid catalyst against *Salmonella typhimurium*.

Organism	Catalysts	Concentration of catalyst (μg/ml)				
		50		100		150
		Zone of inhibition (mm)				
*Salmonella typhimurium*	**rGO-PANI**_**(80:20)**_**/Pd:Au**_**(1:1)**_	**10**	**12**		**14**	
	rGO-PANI_(80:20)_/Pd:Au_(1:2)_	7	8		10	
	rGO-PANI_(80:20)_/Pd:Au_(2:1)_	8	10		11	
	Streptomycin		16			

### Antifungal activity studies

**Figure 16:**
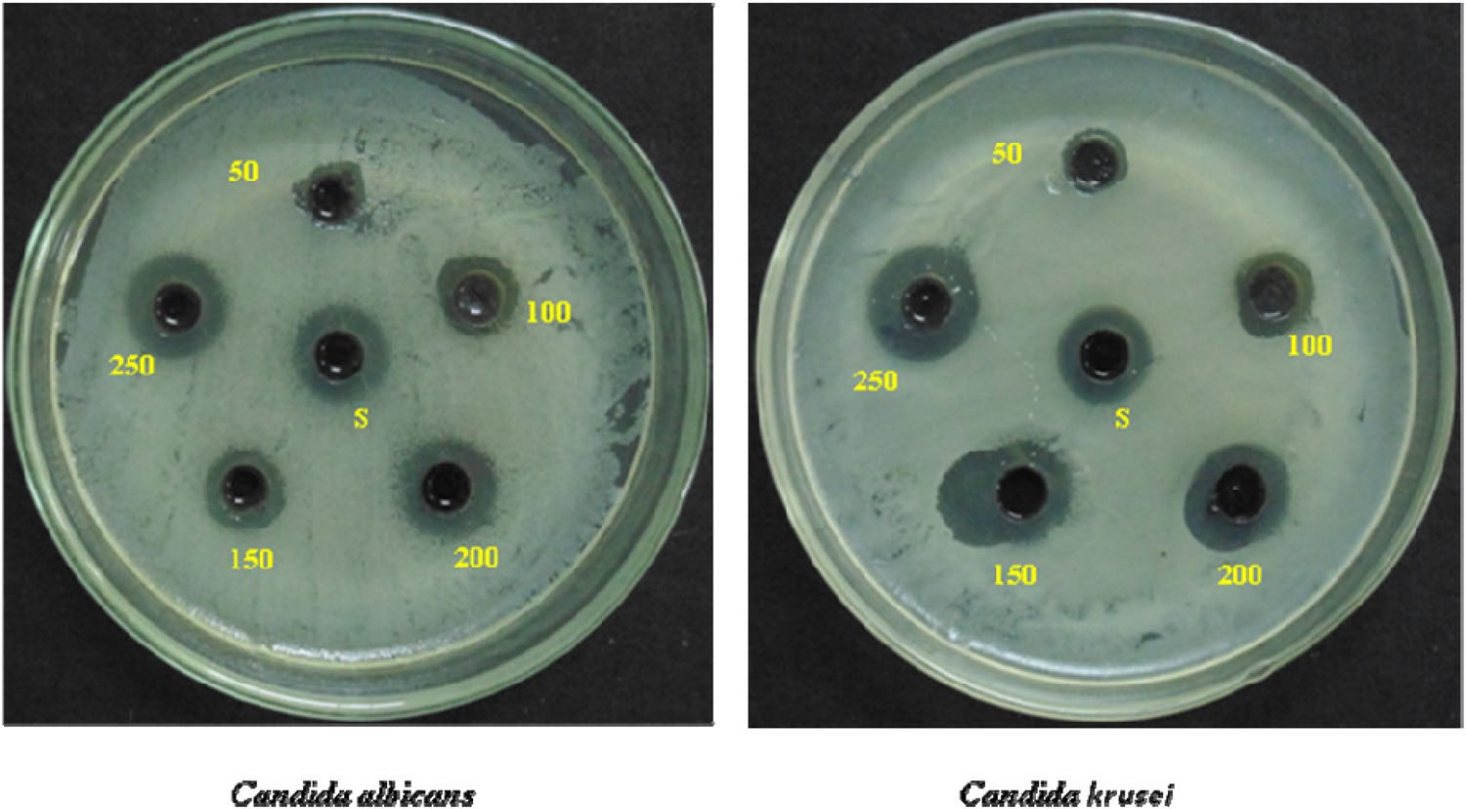
Antifungal activity of an optimized rGO-PANI_(80:20)_ supported Pd:Au_(1:1)_ bimetallic nanocomposite hybrid catalyst and Amphotericin are used as standard drug.

**Figure 17:**
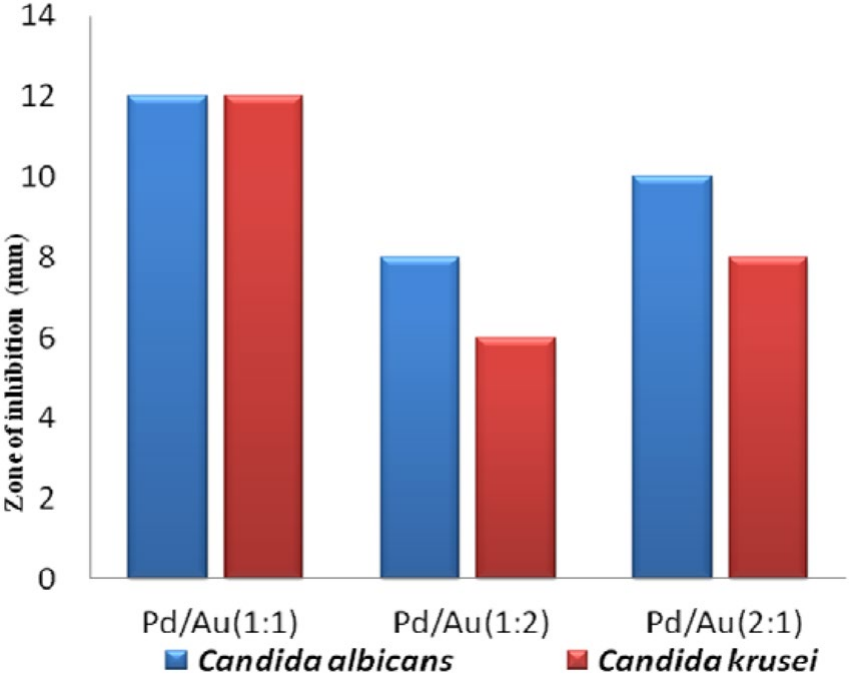
Antifungal activity of an optimized rGO-PANI_(80:20)_ supported Pd:Au_(1:1, 1:2, 2:1)_ bimetallic nanocomposite hybrid catalysts.

An optimized rGO-PANI_(80:20)_ supported Pd:Au_(1:1, 1:2, 2:1)_ bimetallic nanocomposite hybrid catalysts were also examined for their antifungal activity against *Candida albicans* and *Candida kruesi*. The standard drug amphotericin b (30 µg/mL) was used as an antifungal positive control. The antifungal activity of the catalyst was evaluated by measuring the zone of inhibition. The antifungal activity of the rGO-PANI_(80:20)_ supported Pd:Au_(1:1)_ hybrid catalyst increases with increasing the dosage of hybrid catalyst (50, 100, 150, 200 and 250 *μ*L) against both *candida* as shown in Fig. 16. The overall antifungal activities of all the bimetallic nanocomposite hybrid catalysts are shown in Fig. 17. Among them, the nanocomposite bimetallic hybrid catalyst [rGO-PANI_(80:20)_/Pd:Au_(1:1)_] exhibited excellent antifungal activities against human pathogens such as *Candida albicans* and *candida kruesi* at *a* higher dosage than the standard antifungal drug. Therefore, the overall antifungal activity was represented from the higher-order as follows; rGO-PANI_(80:20)_/Pd:Au_(1:1)_ > rGO-PANI_(80:20)_/Pd:Au_(2:1)_ > rGO-PANI_(80:20)_/Pd:Au_(1:2)_. As a result, the rGO-PANI_(80:20)_/Pd:Au_(1:1)_ is chosen as the best potential catalyst with remarkable antimicrobial activity. Interestingly, it is the first report in this combination to examined the antifungal activity.

## Conclusion

We have demonstrated a facile and an efficient route for synthesizing mono (Pd) and bimetallic Pd: Au_(1:1, 1:2, 2:1)_ nanoparticles anchored over rGO-PANI composites by a simple chemical reduction method. The catalytic activities of all the prepared bare GO, rGO, rGO-PANI composite and rGO-PANI supported mono (Pd) & bimetallic Pd: Au_(1:1, 1:2, 2:1)_ nanocomposite hybrid catalysts were studied towards the reduction of different nitroaromatic compounds, organic dyes, and heavy metal ion. Among them, an optimized rGO-PANI_(80:20)_ supported mono (Pd) and bimetallic Pd/Au_(1:1)_ nanocomposite hybrid catalysts showed excellent stability and an efficient catalytic activity towards the reduction of different nitroaromatic compounds, organic dyes and heavy metal ion than that of other composites. In addition to this, the bimetallic nanocomposite hybrid catalyst [rGO-PANI_(80:20)_/Pd: Au_(1:1)_] exhibited antibacterial activity against *S. aureus* and *S. Typhi* as well as fungus of both *Candida albicans* and *Candida kruesi*. The antibacterial and antifungal activity of the catalysts strongly dependent on the initial concentration of them and their inoculation time. We hope, the present findings may open up a new and environmentally benign avenue in the development of noble bimetallic nanocomposite catalysts in the future.

## Supplementary information

Effect of Hybrid mono/bimetallic Nanocomposites for an enhancement of Catalytic and Antimicrobial Activities.
