# Supplementary material for: Effect of Hybrid mono/bimetallic Nanocomposites for an enhancement of Catalytic and Antimicrobial Activities
Source: Sci Rep. 2020 Feb 13;10:2586. doi: 10.1038/s41598-020-59491-5 (PMC7018773; doi:10.1038/s41598-020-59491-5)
Supplement: Supplementary file 1 — Effect of Hybrid mono/bimetallic Nanocomposites for an enhancement of Catalytic and Antimicrobial Activities. [file 41598_2020_59491_MOESM1_ESM.doc]

**Supporting Information**

**Effect of Hybrid mono/bimetallic Nanocomposites for an enhancement of Catalytic and Antimicrobial Activities**

**Kuppan Sivaranjan^1*^, Osaimany Padmaraj^2^, Jayadevan Santhanalakshmi^3*^, Malairaj Sathuvan^4^, Anbazhagan Sathiyaseelan^4^ and Suresh Sagadevan^5^***

^1^Department of Chemistry, Faculty of Science and Mathematics, Universiti Pendidikan Sultan Idris, Tanjung Malim, Perak-35900, Malaysia

^2^Department of Nuclear Physics, University of Madras, Guindy Campus, Chennai-600 025, Tamil Nadu, India

^3^Department of Physical Chemistry, University of Madras, Guindy Campus, Chennai-600 025, Tamil Nadu, India.

^4^Centre for Advanced Studies in Botany, University of Madras, Guindy Campus,Chennai-600 025, Tamil Nadu, India

^5^Nanotechnology & Catalysis Research Centre, University of Malaya, Kuala Lumpur 50603, Malaysia

***E-mail: kksivaranjan@gmail.com,** [**drjslakshmi06@yahoo.co.in**](mailto:drjslakshmi06@yahoo.co.in)**,**[**drsureshnano@gmail.com**](mailto:drsureshnano@gmail.com)

**1. Characterization techniques**

The microscopic investigation are used morphology and shape of rGO-PANI supported mono and bimetallic NPs were characterized by Scanning electron microscopy (SEM-HITACHI, S-3400 N model instrument), Filed emission Scanning electron microscopy (FESEM- SU6600, HITACHI model), the present of elements and functional group analyses by energy-dispersive x-ray spectroscopy (EDAX), high-resolution transmission electron microscopy (HRTEM-FEI-TECNAI G2, model-T-30-S-twin) and the crystal structures of the mono bimetallic NPs were characterized by X-ray diffraction (XRD-Bruker D8 Advance X-ray diffraction system with Cu Kα1 radiation). The electronic structures of the synthesized rGO-PANI supported mono and bimetallic NPs were characterized by X-ray photoelectron spectroscopy (XPS-omicron nanotechnology, ESCA-14 model instrument). The functional group analyses were characterized by Fourier transfer-infrared spectroscopy (FT-IR-Brukertenson 27 infrared spectrometer). The catalytic reduction reaction was monitored by UV-Visible spectroscopy (UV-Vis-UV-Visible techcom 8500 double beam spectrophotometer). The synthesized NPs interracial interaction between the graphene and polyaniline by characterized Raman spectroscopy (Raman-Nanophoton- Raman 11 model instrument).

**S1. SEM images of pure and three different ratios of rGO-PANI_(80:20, 50:50, 10:90)_ composites**

1.
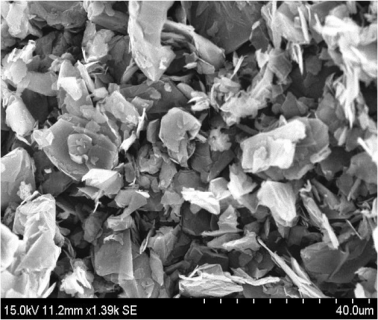
 **B)**
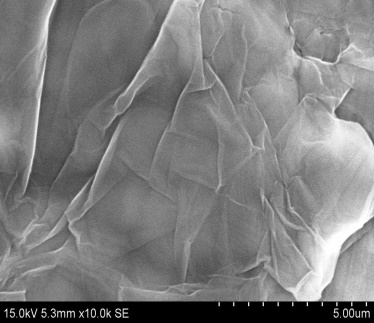
 **C)**
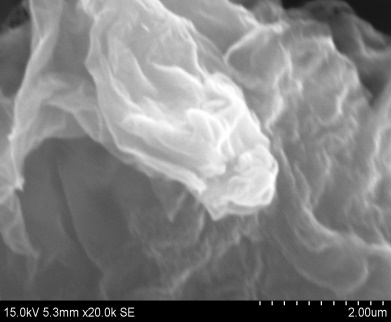

2.
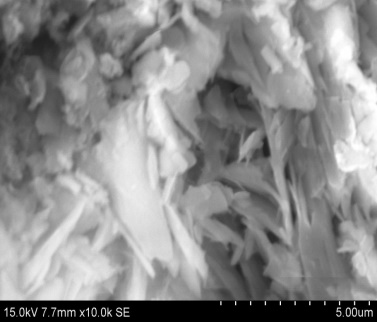
 **E)**
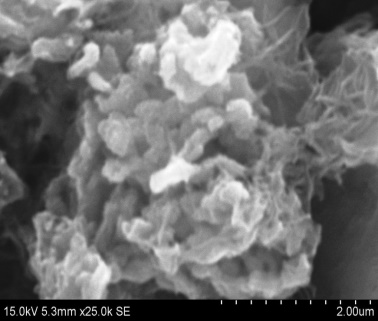
 **F)**
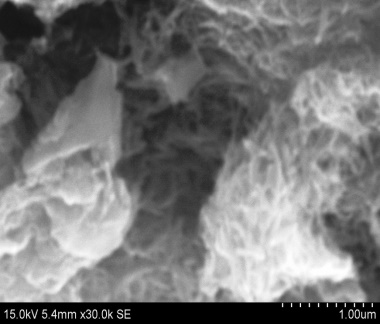


**G)**
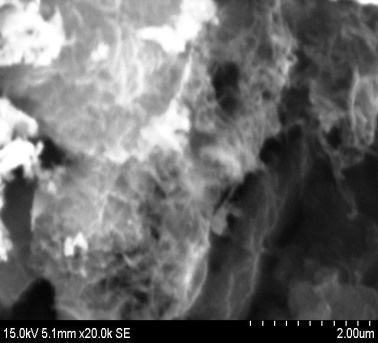


***Figure S1*** SEM images of A) Graphite B) GO C) rGO D) PANI E) rGO-PANI_(80:20)_ F) rGO-PANI_(50:50)_ and G) rGO-PANI_(10:90)_ nanocomposites.

**S2. HR-TEM images of pure and three different ratios of rGO-PANI_(80:20, 50:50, 10:90)_ composites.**

**
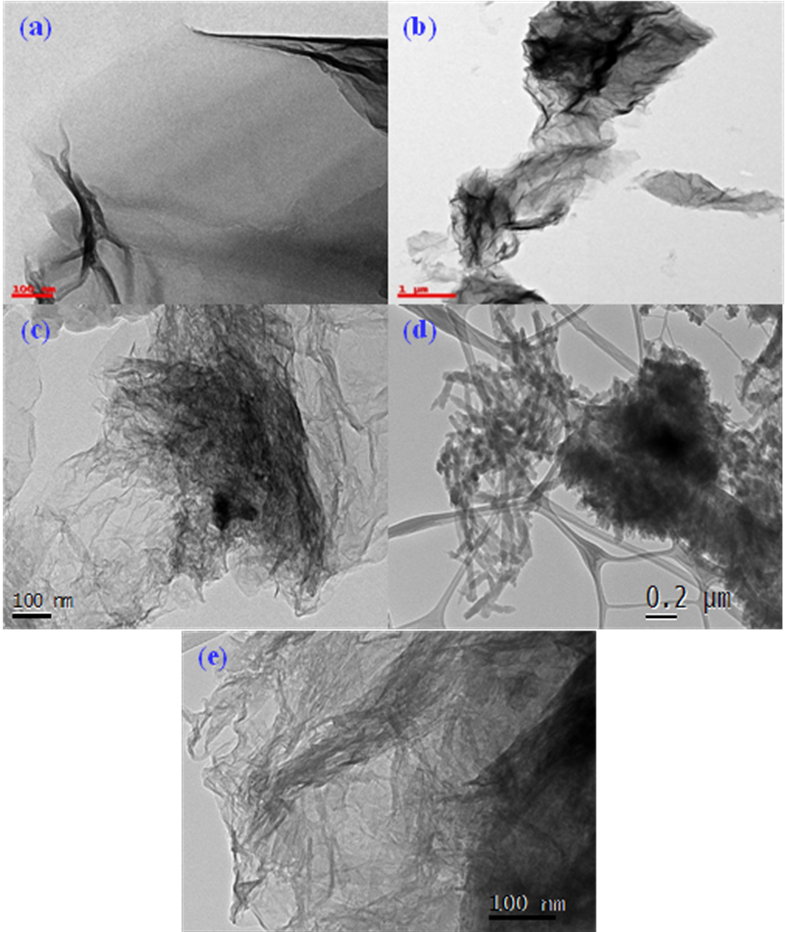
**

***Figure S2*** HR-TEM images of (a) GO; (b) rGO; (c) rGO-PANI_(80:20)_; (d) rGO-PANI_(50:50)_ and (e) rGO-PANI_(10:90)_ nanocomposites.

**S3. FE-SEM and HR-TEM images of three different ratios of rGO-PANI_(80:20, 50:50, 10:90)_ composites supported Pd monometallic nanocomposite [rGO-PANI_(80:20, 50:50, 10:90)_/Pd] hybrid catalysts.**

1.
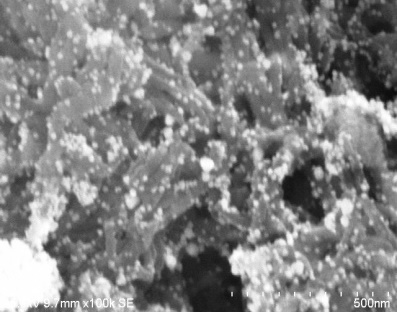
 **B)**
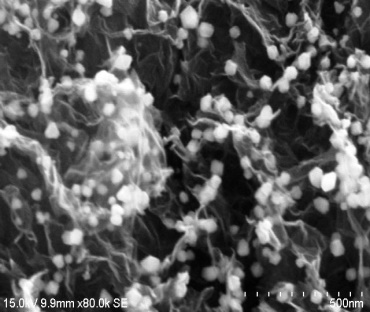
 **C)**
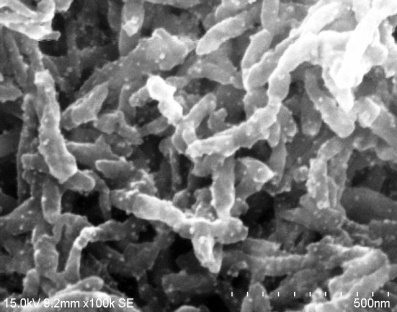

2.
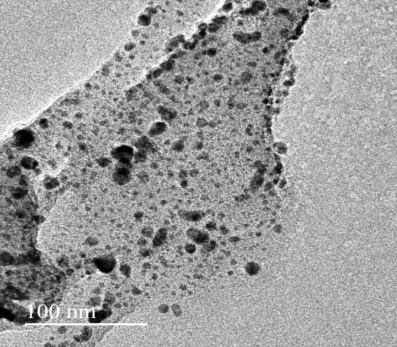
 **E)**
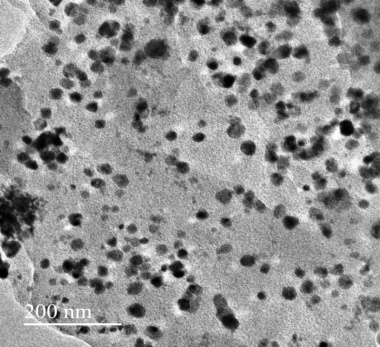
 **F)**
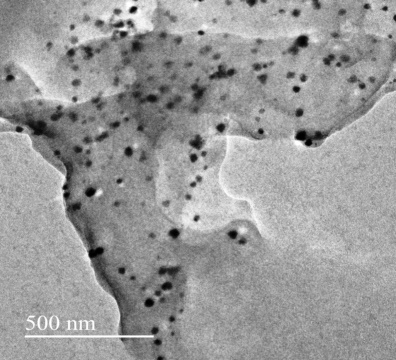


***Figure S3*** FE-SEM images of; A) rGO-PANI_(80:20)_/Pd; B) rGO-PANI_(50:50)_/Pd; C) rGO-PANI_(10:90)_/Pd and HR-TEM images of; D) rGO-PANI_(80:20)_/Pd; E) rGO-PANI_(50:50)_/Pd; F) rGO-PANI_(10:90)_/Pd monometallic nanocomposite hybrid catalysts.

**S4. FE-SEM and HR-TEM images of an optimized rGO-PANI_(80:20)_ composite supported Pd:Au_(1:1, 1:2, 2:1)_ bimetallic nanocomposite [rGO-PANI_(80:20)_/Pd:Au_(1:1, 1:2, 2:1)_] hybrid catalysts.**

1. **
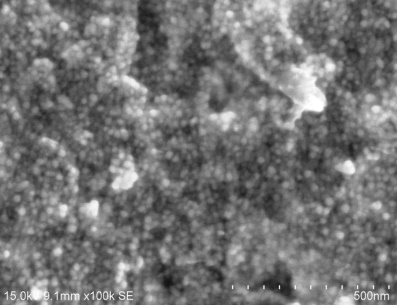
 B)
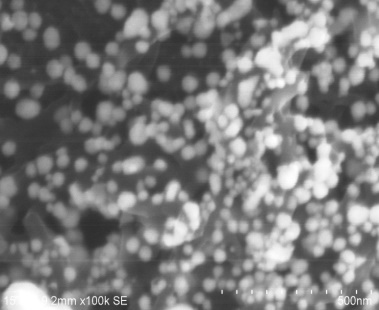
 C)
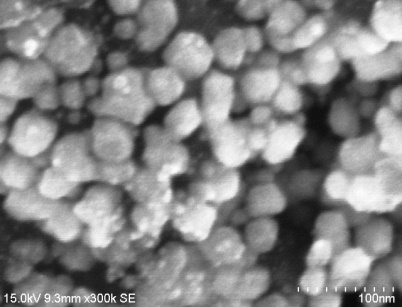
**
2. **
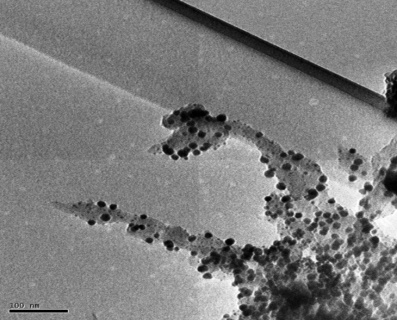
 E)
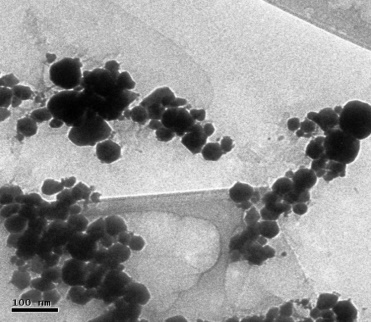
 F)
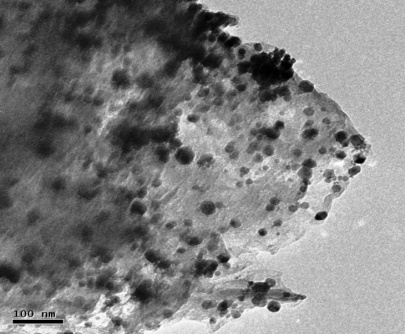
**

***Figure S4*** FE-SEM images of; A) rGO-PANI_(80:20)_/Pd:Au_(1:1)_; B) rGO-PANI_(80:20)_/Pd:Au_(1:2)_; C) rGO-PANI_(80:20)_/Pd:Au_(2:1)_; and HR-TEM images of D) rGO-PANI_(80:20)_/Pd:Au_(1:1)_; E) rGO-PANI_(80:20)_/Pd:Au_(1:2)_; F) rGO-PANI_(80:20)_/Pd:Au_(2:1)_ bimetallic nanocomposite hybrid catalysts.

**S5. EDX spectra of three different ratios of rGO-PANI_(80:20, 50:50, 10:90)_ composites supported Pd monometallic nanocomposite [rGO-PANI_(80:20, 50:50, 10:90)_/Pd] hybrid catalysts.**

**
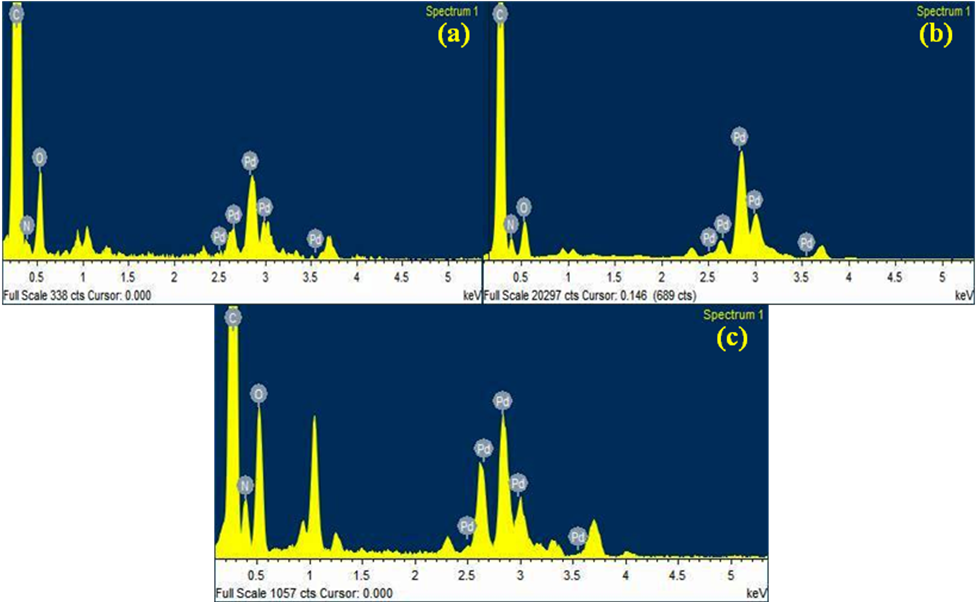
**

***Figure S5*** EDX spectra of (a) rGO-PANI_(80:20)_/Pd (b) rGO-PANI_(50:50)_/Pd and (c) rGO-PANI_(10:90)_/Pd monometallic nanocomposite hybrid catalysts.

**S6. EDX spectra of an optimized rGO-PANI_(80:20)_ composite supported Pd:Au_(1:1, 1:2, 2:1)_ bimetallic nanocomposite [rGO-PANI_(80:20)_/Pd:Au_(1:1, 1:2, 2:1)_] hybrid catalysts.**

**
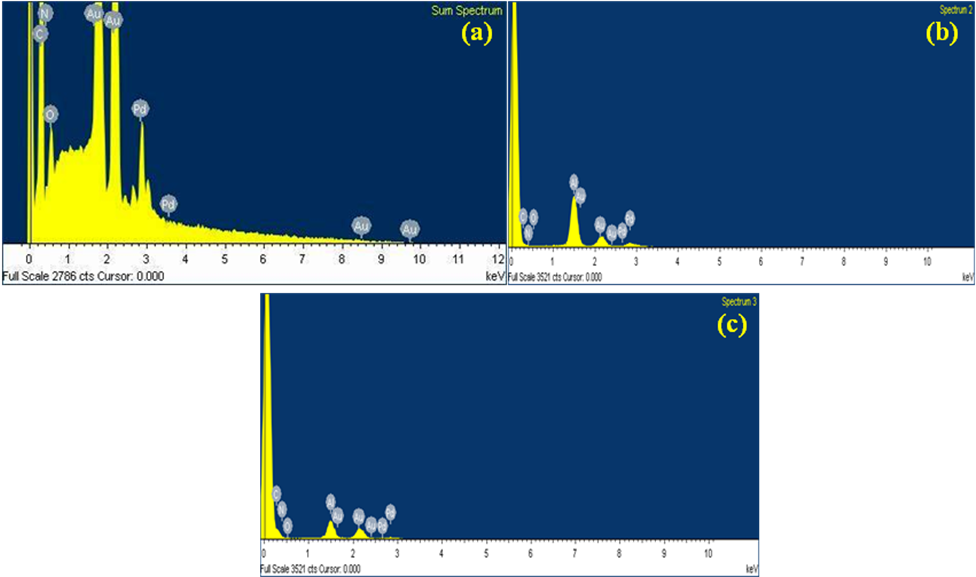
**

***Figure S6*** EDX spectra of (a) rGO-PANI_(80:20)_/Pd:Au_(1:1)_, (b) rGO-PANI_(80:20)_/Pd:Au_(1:2)_ and (c) rGO-PANI_(80:20)_/Pd:Au_(2:1)_ bimetallic nanocomposite hybrid catalyst.

**S7. Elemental mappings of an optimized rGO-PANI_(80:20)_ composite supported Pd:Au_(1:1)_ bimetallic nanocomposite [rGO-PANI_(80:20)_/Pd:Au_(1:1)_] hybrid catalysts.**

**
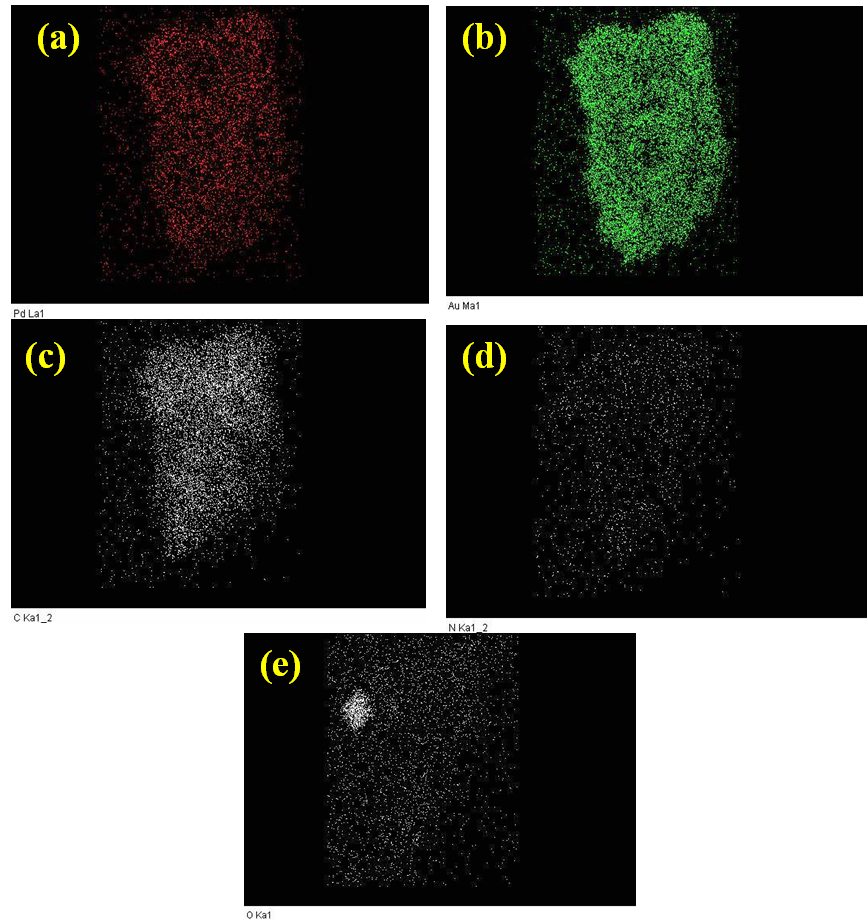
**

***Figure S7*** Elemental mappings of (a) Pd (b) Au (c) C (d) N and (e) O elements in an optimized rGO-PANI_(80:20)_ supported Pd:Au_(1:1)_ bimetallic nanocomposite hybrid catalyst.

**S8(A). UV-Vis spectra of pure and three different ratios of rGO-PANI_(80:20, 50:50, 10:90)_ composites.**

***
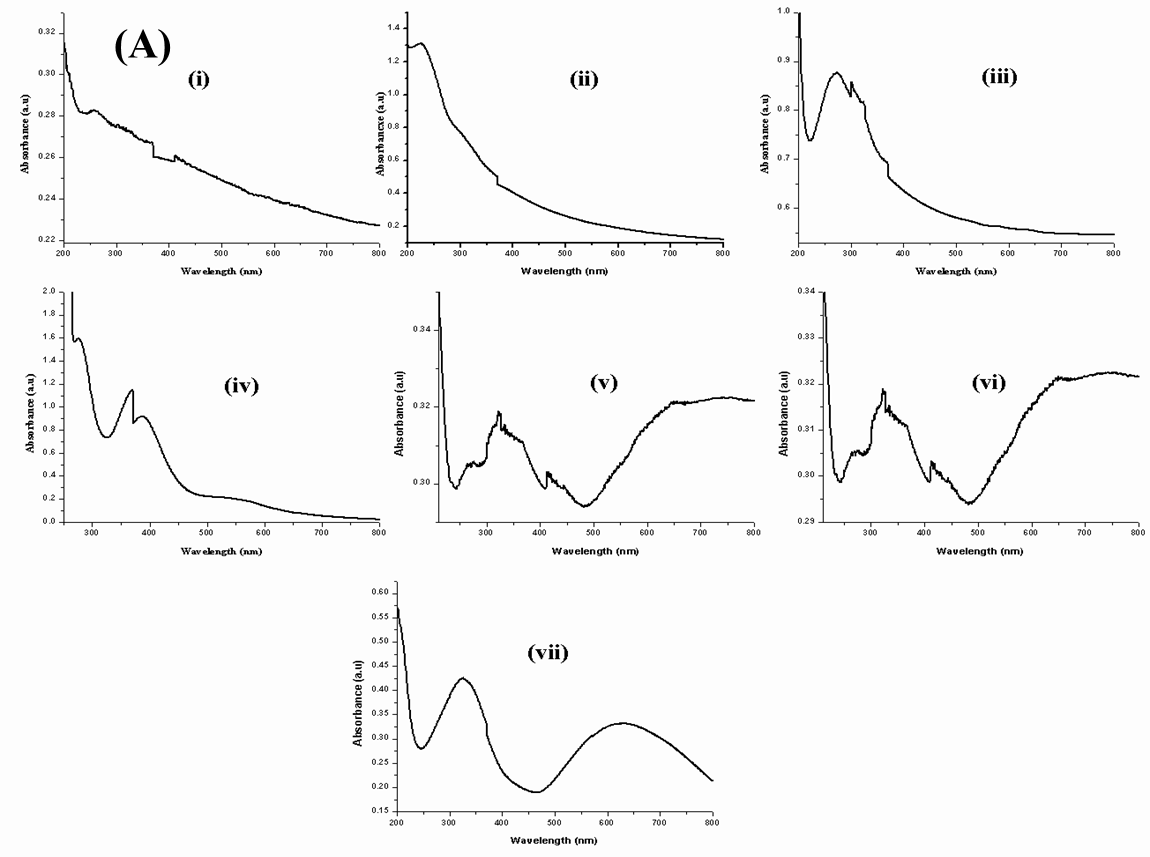
***

***Figure S8(*A)** UV-Visible spectra of (i) graphite, (ii) GO, (iii) rGO, (iv) PANI, (v) rGO-PANI_(80:20)_, (vi) rGO-PANI_(50:50)_ and (vii) rGO-PANI_(10:90)_ composites.

**S8(B). UV-Vis spectra of three different ratios of rGO-PANI_(80:20, 50:50, 10:90)_ composites supported Pd monometallic [rGO-PANI_(80:20, 50:50, 10:90)_/Pd] and an optimized rGO-PANI_(80:20)_ composite supported Pd:Au_(1:1, 1:2, 2:1)_ bimetallic nanocomposite [rGO-PANI_(80:20)_/Pd:Au_(1:1, 1:2, 2:1)_] hybrid catalysts.**

***
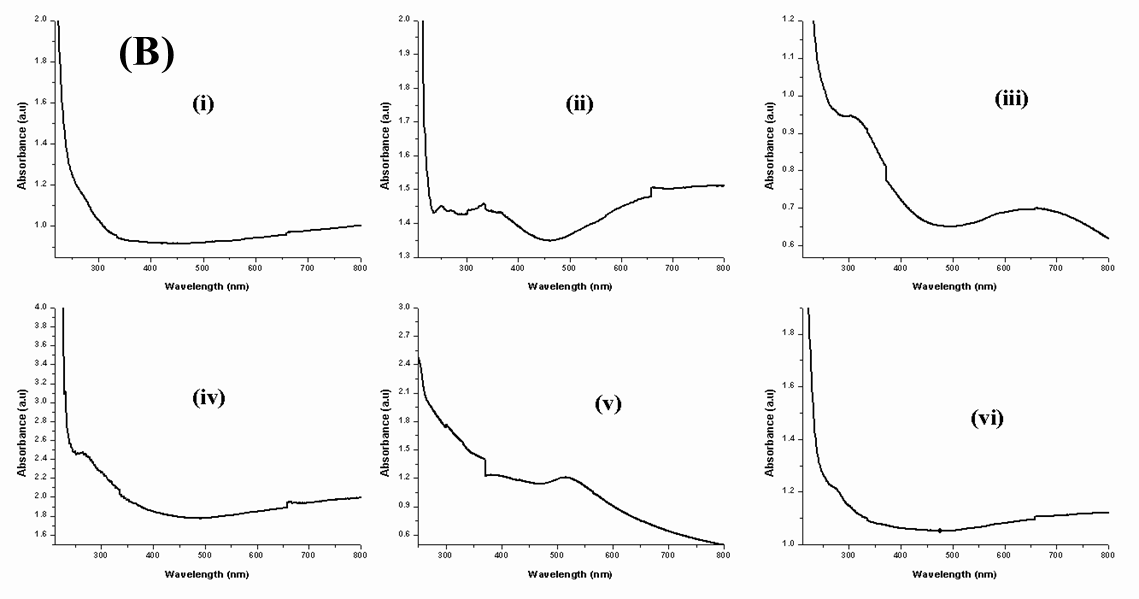
***

***Figure S8 (*B)** UV-Visible spectrum of (i) rGO-PANI_(80:20)_/Pd, (ii) rGO-PANI_(50:50)_/Pd, (iii) rGO-PANI_(10:90)_/Pd, (iv) rGO-PANI_(80:20)_/Pd:Au_(1:1)_, (v) rGO-PANI_(80:20)_/Pd:Au_(1:2)_ and (vi) rGO-PANI_(80:20)_/Pd:Au_(2:1)_ bimetallic nanocomposite hybrid catalysts..

**S9. Time-dependent UV–vis spectra for the reduction of p-Nitrophenol using NaBH_4_ in the presence of an optimized rGO-PANI_(80:20)_ composites supported Pd monometallic nanocomposite [rGO-PANI_(80:20)_/Pd] hybrid catalysts.**

**
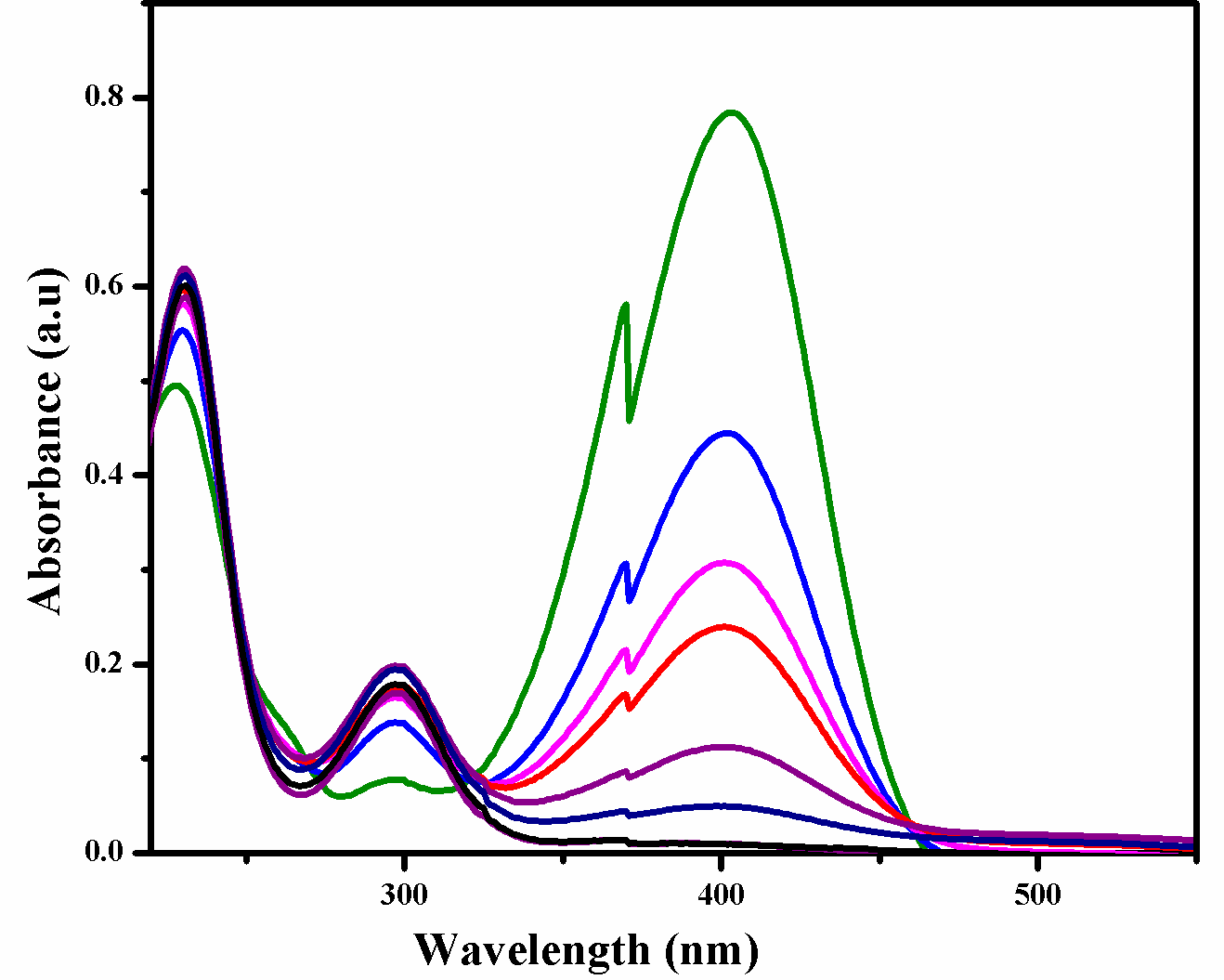
**

***Figure S9*** Time-dependent UV–vis spectra for the reduction of p-Nitrophenol using NaBH_4_ in the presence of rGO-PANI_(80:20)_/Pd monometallic nanocomposite hybrid catalyst.
